# Exhaled volatilome analysis as a useful tool to discriminate asthma with other coexisting atopic diseases in women of childbearing age

**DOI:** 10.1038/s41598-021-92933-2

**Published:** 2021-07-05

**Authors:** Rosa A. Sola-Martínez, Gema Lozano-Terol, Julia Gallego-Jara, Eva Morales, Esther Cantero-Cano, Manuel Sanchez-Solis, Luis García-Marcos, Pedro Jiménez-Guerrero, José A. Noguera-Velasco, Manuel Cánovas Díaz, Teresa de Diego Puente, the NELA study group, M. E. Candel-Torralba, L. Garcia-Marcos, M. J. Gimenez-Banon, A. Martinez-Torres, E. Morales, V. Perez-Fernandez, M. Sanchez-Solis, A. Nieto, M. T. Prieto-Sanchez, M. Sanchez-Ferrer, L. Fernandez-Palacios, V. P. Gomez-Gomez, C. Martinez-Gracia, P. Peso-Echarri, G. Ros-Berruezo, M. Santaella-Pascual, A. Gazquez, E. Larque, M. T. Pastor-Fajardo, M. Sanchez-Campillo, A. Serrano-Munuera, M. Zornoza-Moreno, P. Jimenez-Guerrero, E. Adomnei, J. J. Arense-Gonzalo, J. Mendiola, F. Navarro-Lafuente, A. M. Torres-Cantero, C. Salvador-Garcia, M. Segovia-Hernández, G. Yagüe-Guirao, P. L. Valero-Guillén, F. V. Aviles-Plaza, J. Cabezas-Herrera, A. Martinez-Lopez, M. Martinez-Villanueva, J. A. Noguera-Velasco, E. Cantero-Cano, A. Franco-Garcia, A. M. Garcia-Serna, T. Hernandez-Caselles, E. Martin-Orozco, M. Norte-Muñoz, M. Cánovas Díaz, T. de Diego Puente, J. M. Pastor, R. A. Sola-Martínez, A. Esteban-Gil, J. T. Fernández-Breis, M. V. Alcántara, S. Hernández, C. López-Soler

**Affiliations:** 1 Department of Biochemistry and Molecular Biology B and Immunology, University of Murcia, Murcia, Spain; 2 Biomedical Research Institute of Murcia, IMIB-Arrixaca, Murcia, Spain; 3 Department of Public Health Sciences, University of Murcia, Murcia, Spain; 4 Respiratory and Allergy Units, Arrixaca Children’s University Hospital, University of Murcia, Murcia, Spain; 5 Department of Paediatrics, University of Murcia, Murcia, Spain; 6 Network of Asthma and Adverse and Allergy Reactions (ARADyAL), Health Institute Carlos III, Madrid, Spain; 7 Regional Atmospheric Modelling Group, Department of Physics, University of Murcia, Murcia, Spain; 8 Molecular Therapy and Biomarkers Research Group, Clinical Analysis Service, University Clinical Hospital “Virgen de la Arrixaca”, University of Murcia, Murcia, Spain; 9 Obstetrics & Gynaecology Service, “Virgen de la Arrixaca” University Clinical Hospital, University of Murcia, Murcia, Spain; 10 Food Science and Technology Department, Veterinary Faculty of Veterinary, University of Murcia, Murcia, Spain; 11 Department of Physiology, Faculty of Biology, University of Murcia, Campus Mare Nostrum, Murcia, Spain; 12 Microbiology Service, General University Hospital Consortium, University of Valencia, Murcia, Spain; 13 Microbiology Service, University Clinical Hospital “Virgen de la Arrixaca”, University of Murcia, Murcia, Spain; 14 Microbiology and Genetics Department, University of Murcia, Murcia, Spain; 15 Department of Informatics and Systems, University of Murcia, Murcia, Spain; 16 Paediatric and Adolescent Clinical Psychology University Research Group (GUIIA-PC), University of Murcia, Murcia, Spain; 17 Foundation for Healthcare Training & Research of the Region of Murcia (FFIS), Murcia, Spain

**Keywords:** Biochemistry, Computational biology and bioinformatics, Systems biology, Biomarkers, Diseases, Medical research

## Abstract

The prevalence of asthma is considerably high among women of childbearing age. Most asthmatic women also often have other atopic disorders. Therefore, the differentiation between patients with atopic diseases without asthma and asthmatics with coexisting diseases is essential to avoid underdiagnosis of asthma and to design strategies to reduce symptom severity and improve quality of life of patients. Hence, we aimed for the first time to conduct an analysis of volatile organic compounds in exhaled breath of women of childbearing age as a new approach to discriminate between asthmatics with other coexisting atopic diseases and non-asthmatics (with or without atopic diseases), which could be a helpful tool for more accurate asthma detection and monitoring using a noninvasive technique in the near future. In this study, exhaled air samples of 336 women (training set (n = 211) and validation set (n = 125)) were collected and analyzed by thermal desorption coupled with gas chromatography-mass spectrometry. ASCA (ANOVA (analysis of variance) simultaneous component analysis) and LASSO + LS (least absolute shrinkage and selection operator + logistic regression) were employed for data analysis. Fifteen statistically significant models (p-value < 0.05 in permutation tests) that discriminated asthma with other coexisting atopic diseases in women of childbearing age were generated. Acetone, 2-ethyl-1-hexanol and a tetrahydroisoquinoline derivative were selected as discriminants of asthma with other coexisting atopic diseases. In addition, carbon disulfide, a tetrahydroisoquinoline derivative, 2-ethyl-1-hexanol and decane discriminated asthma disease among patients with other atopic disorders. Results of this study indicate that refined metabolomic analysis of exhaled breath allows asthma with other coexisting atopic diseases discrimination in women of reproductive age.

## Introduction

Asthma is a chronic disease that involves an enormous economic cost for the healthcare systems of nations. Moreover, asthma prevalence worldwide has increased considerably in recent years^1^. Previous studies have suggested that factors associated with changes in lifestyles and environmental contaminants during pregnancy influence the risk of asthma or other atopic conditions in children^2,3^. Atopic conditions usually include such diseases as atopic dermatitis, asthma, food allergy or allergic rhinitis. All these diseases are strongly associated with each other, and the manifestation of one often involves the onset of the others^4,5^. Thus, asthma frequently also coexists with other atopic diseases^6^. To date, many studies have shown the progression of atopic disorders from atopic dermatitis in infants to allergic rhinitis and asthma in children^7–9^. Furthermore, the prevalence of asthma differs between males and females: although asthma is more prevalent in males during childhood, the prevalence is higher in females in adulthood. Specifically, the prevalence is rather high among women of childbearing age, and symptoms tend to be more severe^10,11^.

**Figure 1:**
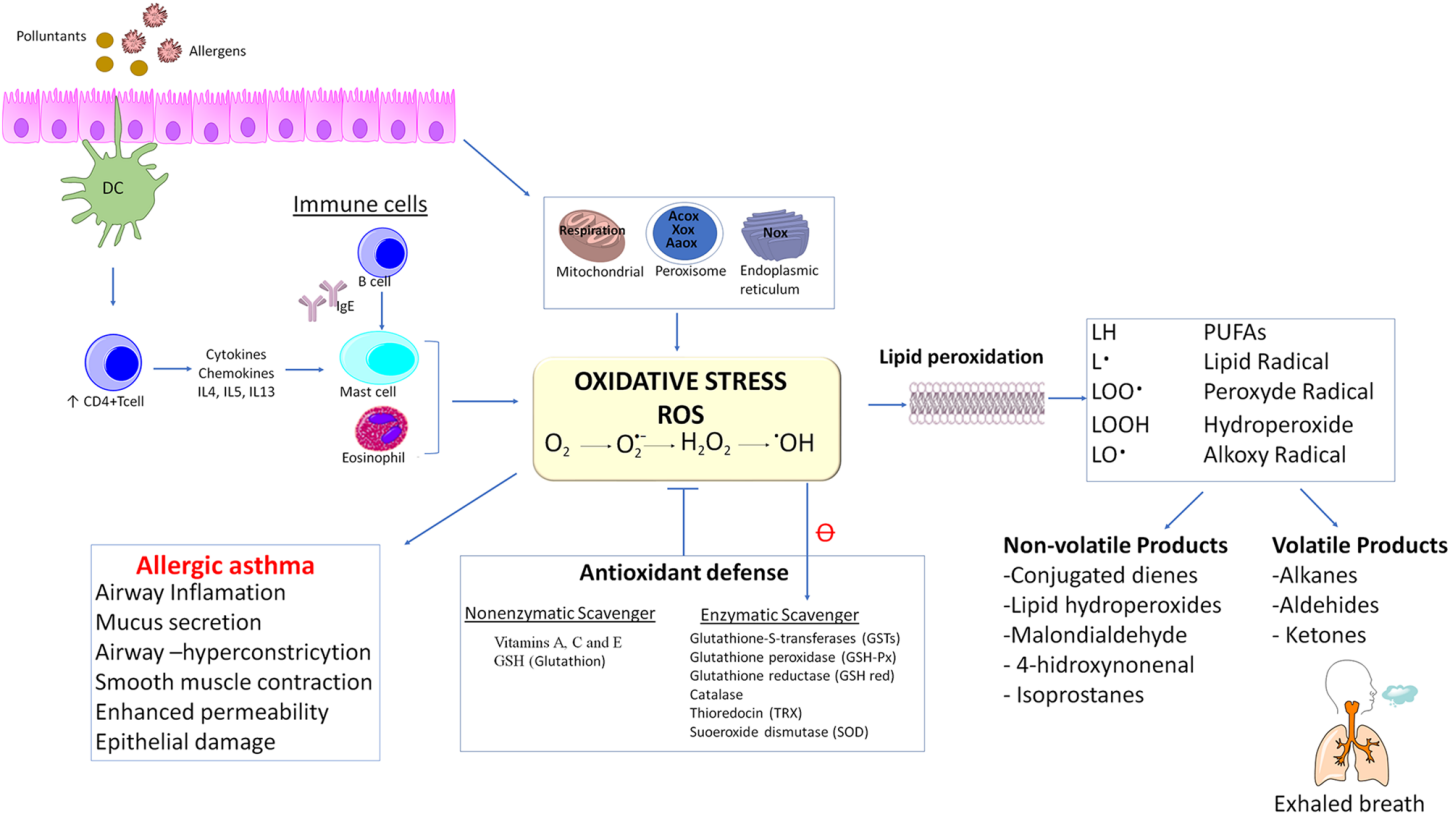
Lipid peroxidation and volatile organic compounds production in allergic asthma. *PUFAs* polyunsaturated fatty acids, *ROS* reactive oxygen species, *Nos* NADPH oxidase, *Acox* AcilCoA-oxidase, *AaOx* amino acid oxidase.

Airway inflammation is the most typical characteristic of asthma. Increased oxidative stress plays an important role in airway inflammation^12^ and is often linked to enhanced ROS (reactive oxygen species) production or malfunction of antioxidant defenses^13,14^. ROS generation can affect DNA, lipids, proteins and carbohydrates^15^. ROS may be derived from endogenous sources, such as cellular organelles (mitochondria, peroxisomes or the endoplasmic reticulum), among others; allergens or environmental pollutants can also promote ROS production^12,14,16^. In allergic asthma (the most prevalent asthma), the presence of allergens generates ROS via a complex activation mechanism that involves mainly dendritic cell (DC) activation, CD4 + T cell activation, interleukin production, IgE production by B cells, and activation of mast cells and eosinophils^12^ (Fig. 1).

Currently, clinical history and spirometry are the most reliable methods for asthma diagnosis^17,18^. However, they do not allow for assessment of airway inflammation. Although blood eosinophil count has been suggested as a useful tool for asthma diagnosis, levels can be influenced by several factors^19,20^. Techniques such as induced sputum, bronchoscopy with bronchoalveolar lavage (BAL) and biopsy are suitable for airway inflammation and oxidative stress assessment, but only induced sputum is a noninvase technique^21,22^. In this sense, scientific community is searching for new alternative noninvasive techniques or biomarkers for asthma phenotypes identification, treatment monitoring, exacerbations prediction, differential diagnosis of other pathologies with similar symptoms and personalized diagnosis when patients suffer from comorbidities such as rhinosinusitis or other atopic disorders^6,22,23^.

Exhaled breath analysis is a noninvasive approach for assessing inflammation of the airway and oxidative stress^21^. In fact, several volatile organic compounds (VOCs) are produced during oxidative stress and lipid peroxidation^24^. The determination of exhaled fractional nitric oxide (FeNO) has provided additional information on airway inflammation as a diagnostic tool for atopic asthma in numerous studies. Nevertheless, it is not useful for non-allergic asthma, and changes in FeNO levels can be indicative of several disorders other than asthma^21,25,26^. Hundreds of different volatile organic compounds can be detected in exhaled breath^27^, and VOCs in exhaled breath are analyzed mainly by technologies based on mass spectrometry or on sensor arrays such as the electronic nose (e-NOSE)^23,28^ Currently, gas chromatography coupled with mass spectrometry (GC/MS) is one of the most suitable techniques because it enables VOC identification in exhaled breath samples with high sensitivity in the ppb range^29^. VOCs measured in exhaled breath may be due to exposure to exogenous contaminants (exposomes) or have an endogenous source, whereby they are produced by human metabolism or even gut bacteria^30^. For all these reasons, analysis of VOCs in exhaled breath has been suggested as an emerging approach for the prediction, diagnosis, and monitoring of asthma^22,31^.

Unfortunately, this strategy is still in the exploratory phase, and several aspects have to be improved for its introduction in a clinical context^29,32,33^. Therefore, the best metabolomic practices are essential to overcome this initial phase, for instance, conducting studies that involve large numbers of subjects, assessment of environmental influences on breath samples, implementation of reproducible and transparent data preprocessing workflows and performing a robust data analysis, among others^29,33^. In this regard, the identification of VOCs as possible biomarkers of diseases requires an adequate data analysis. A large volume of research has considered the creation of predictive models for clinical data; however, much existing literature reports attractive results not validated in an independent set for checking model interpretability and generalizability^29,33^.

This paper is focused on VOC analysis in exhaled breath from women of childbearing age participating in a population-based birth cohort to discriminate between asthmatics with other coexisting atopic diseases and non-asthmatics (with or without atopic diseases). In this study, well-established metabolomics practices were implemented. In this sense, we have carried out a modeling process with a cross-validation on training set and an external validation using a separate test set which guarantees the generalizability of the model to apply to unknown data. In addition, data compiled in the European Health Survey in Spain 2014 (EHSS) and in the Spanish National Health Survey 2017 were statistically analysed in order to identify factors associated with asthma disease.

Methodological development of exhaled breath analysis can greatly enhance our ability to understand the heterogeneity of asthma with other coexisting atopic diseases and atopic disorders without asthma by identifying exhaled volatile organic compounds biomarkers.

## Results

### Analysis of the data collected in health surveys in Spain in recent years

#### Analysis of data collected in the European Health Survey in Spain of 2014 (EHSS-2014)

**Figure 2:**
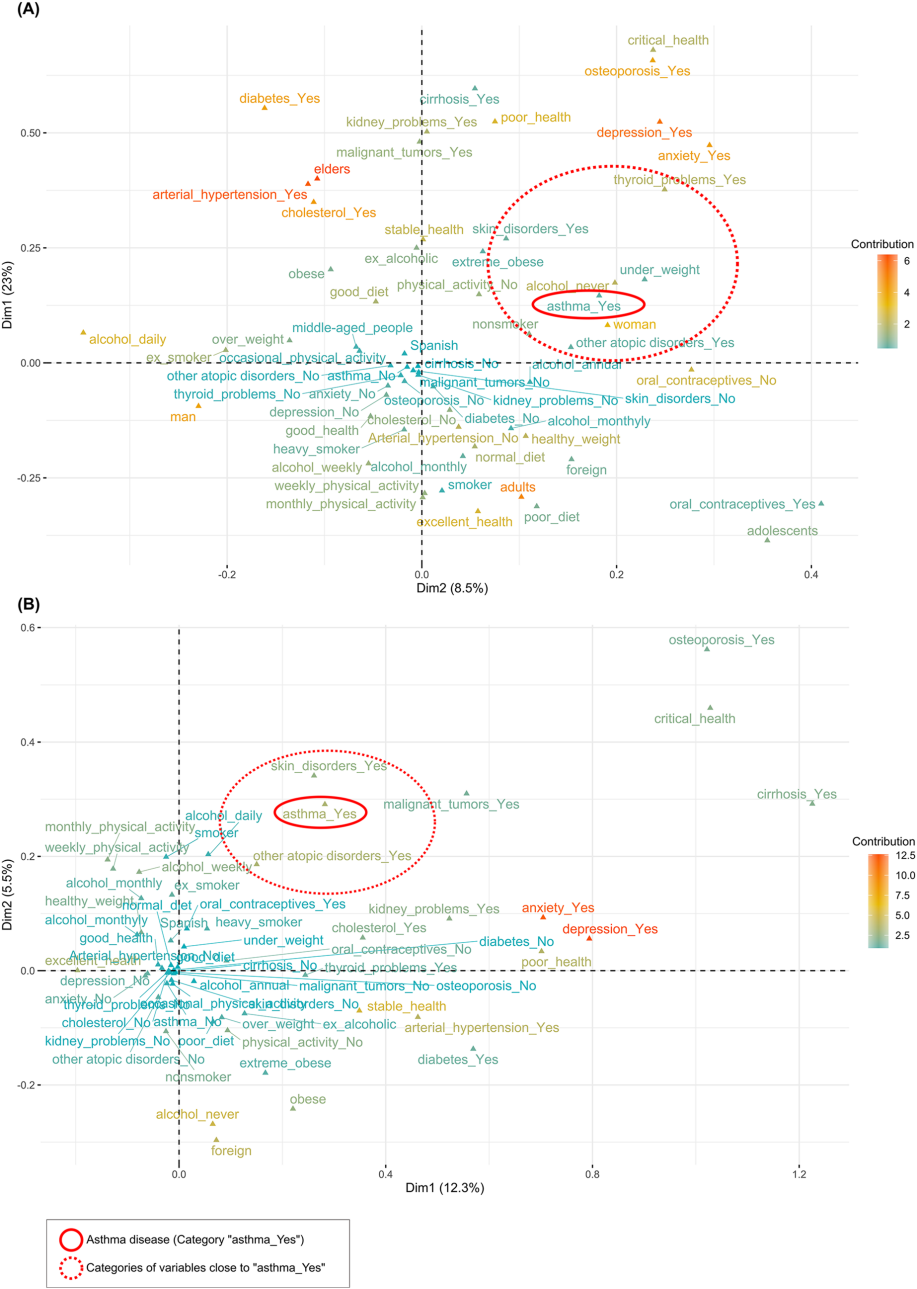
Representation of MCA (multiple corresponde analysis) results performed on data collection from European Health Survey in Spain 2014 (EHSS-2014). (**A**) All data collected from the EHSS-2014. (**B**) Open cohort of 18- to 45-year-old women constructed using the EHSS-2014.

Representation of MCA (multiple correspondence analysis) conducted with the EHSS-2014 data collection is shown in Fig. 2A. The results of chi-square tests and Fisher's exact tests (Supplemental Table S1) showed that the variable "asthma" had a significant relationship with the following variables: age, gender, health status, arterial hypertension, other atopic disorders (allergic rhinitis, atopic dermatitis, allergic conjunctivitis, food allergy or other allergies (excluding asthma)), diabetes, skin disorders, cirrhosis, depression, anxiety disorders, kidney problems, thyroid problems, osteoporosis, Body Mass Index (BMI), physical activity and tobacco. Representation of MCA conducted with an open cohort of 18- to 45-year-old women constructed using EHSS-2014 data collection is shown in Fig. 2B. 6.9% of women of childbearing age were asthmatics. The results of chi-square tests and Fisher's exact tests on data of women of childbearing age (Supplemental Table S1) showed that the variable "asthma" had a significant relationship with the following variables: nationality, health status, arterial hypertension, other atopic disorders, skin disorders, anxiety disorders, cholesterol, BMI and diet.

#### Analysis of data collected in the Spanish National Health Survey of 2017 (ENSE-2017)

Representation of MCA conducted with the ENSE-2017 data collection is shown in Supplemental Fig. S1. The results of chi-square tests and Fisher's exact tests (Supplemental Table S1) showed that the variable "asthma" had a significant relationship with the following variables: age, gender, nationality, arterial hypertension, health status, other atopic disorders, diabetes, skin disorders, cholesterol, cirrhosis, depression, anxiety disorders, kidney problems, malignant tumors, thyroid problems, osteoporosis, BMI, tobacco and alcohol. Representation of MCA conducted with an open cohort of 18- to 45-year-old women constructed using ENSE-2017 data collection is shown in Supplemental Fig. S1. 7.1% of women of childbearing age were asthmatics. The results of chi-square tests and Fisher's exact tests on data of women of childbearing age (Supplemental Table S1) showed that the variable "asthma" had a significant relationship with the following variables: health status, arterial hypertension, other atopic disorders, skin disorders, depression, anxiety disorders and alcohol.

### Subject characteristics: associations between atopic conditions

**Figure 3:**
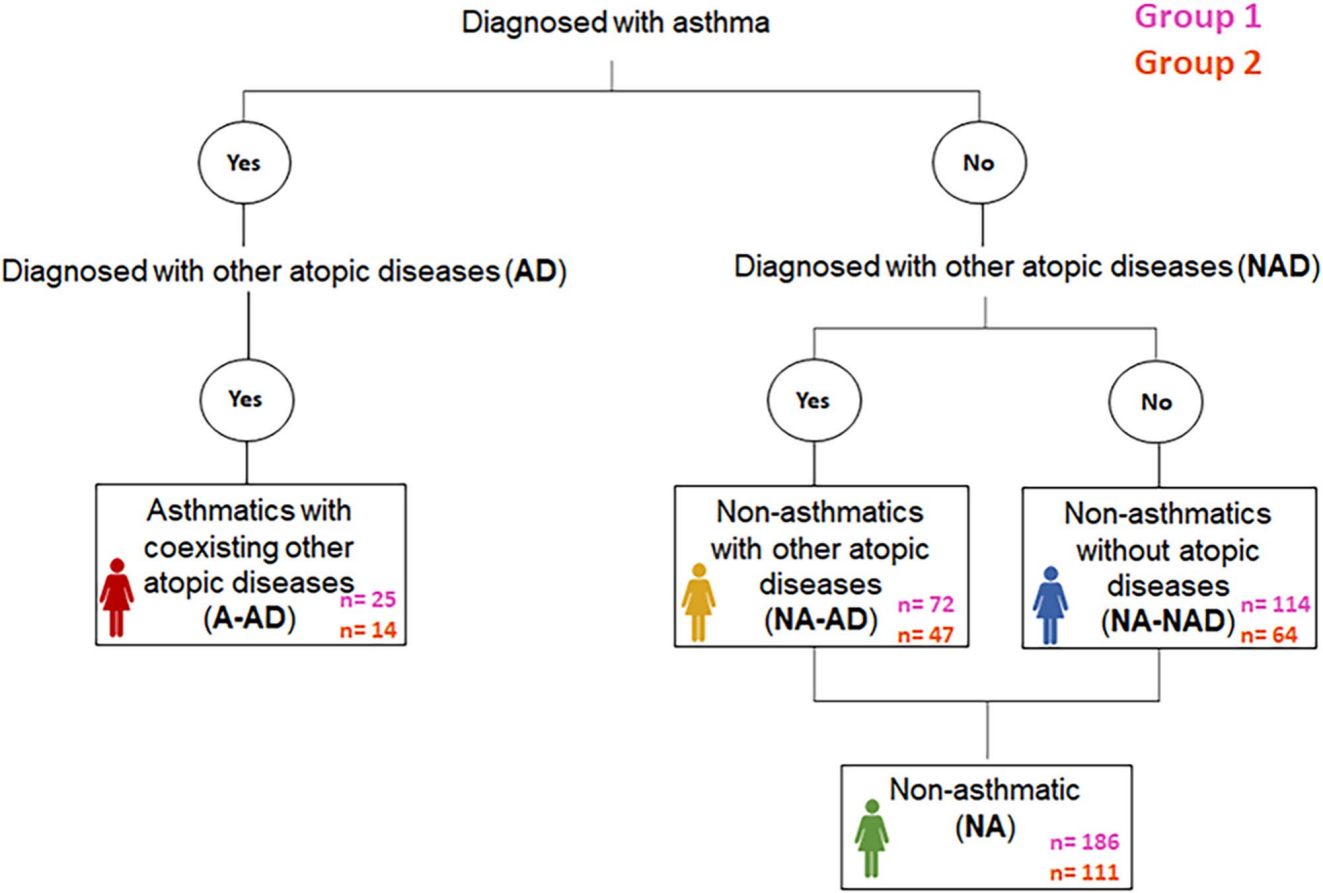
Study flow chart. Other atopic diseases include allergic rhinitis, atopic dermatitis, allergic conjunctivitis, food allergy or drug allergy.

**Figure 4:**
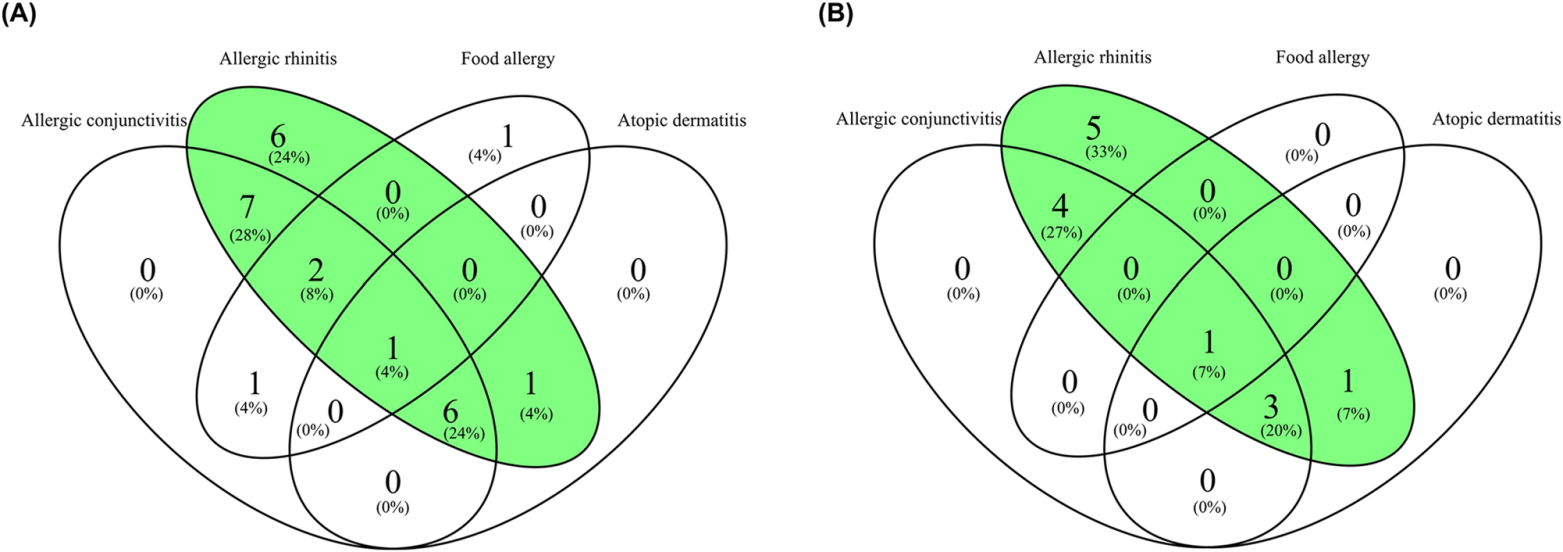
Coexistence of atopic conditions in women with asthma. (**A**) Venn diagram of the coexistence of atopic conditions in women with asthma in Group 1. (**B**) Venn diagram of the coexistence of atopic conditions in women with asthma in Group 2.

A total of 337 women from the NELA (Nutrition and Early Life) cohort participated in the present study. The women were randomly distributed into two groups: Group 1 (n = 211) used as training set and Group 2 (n = 126) used as validation set. Based on asthma and other atopic diseases diagnosed, women in each group were classified into four categories: asthmatics with other coexisting atopic diseases (A-AD), non-asthmatics with other atopic diseases (NA-AD), non-asthmatics without atopic diseases (NA-NAD), and non-asthmatics (NA) (this category includes both non-asthmatics with other atopic disease and non-asthmatics without atopic diseases). Study flow chart is shown in Fig. 3. One woman of Group 2 was excluded for being asthmatic without other atopic diseases. Figure 4 includes two Venn diagrams that show the coexistence of atopic conditions in women with asthma in both groups. Allergic rhinitis and allergic conjunctivitis were the most common coexisting atopic conditions in the women with asthma. The characteristics of the women of Group 1 and Group 2 are shown in Tables 1 and 2, respectively. The percentage of women with allergic rhinitis was higher in A-AD than in NA-AD in both Group 1 and Group 2. The number of females of Group 1 with atopic parental history (parental history of asthma and allergic rhinitis) in NA and NA-NAD was lower than in A-AD. Moreover, in Group 1, percentage of women with parental allergic rhinitis history was higher in NA-AD and NA-NAD. In addition, blood eosinophil count has been found to be significantly higher in A-AD than in NA, NA-AD and NA-NAD in both Group 1 (A-AD vs. NA (p-value = 4.4e-05), A-AD vs. NA-AD (p-value = 8.3e-05), and A-AD vs. NA-NAD (p-value = 0.00014)) and Group 2 (A-AD vs. NA (p-value = 0.0012), A-AD vs. NA-AD (p-value = 0.0086), and A-AD vs. NA-NAD (p-value = 0.00075)) (Supplementary Fig. S2).

**Table 1:** Characteristics of the study population: women of childbearing age (Training set—Group 1).

	Group 1 (211)			
	A-AD	NA	NA-AD	NA-NAD
Subjects n	25	186	72	114
Age (years), mean (range)	32.4 (20–39)	32.9 (20–43)	32.8 (23–41)	33.0 (20–43)
BMI (kg/m^2^) before pregnant, mean (range)	25.23 (19.72–36.81)	24.13 (16.23–42.32)	23.59 (16.65–38.67)	24.48 (16.23–42.32)
**Social class**				
Managers-technicians/skilled/semiskilled-unskilled/unemployed, %	36.0/20.0/20.0/24.0	35.5/18.3/22.0/24.2	34.7/15.2/20.8/29.2	36.0/20.2/22.8/21.1
**Educational level**				
Incomplete secondary or less/complete secondary/university %	28.0/12.0/60.0	19.3/25.3/55.4	25.0/27.8/47.2	15.8/23.7/60.5
Smoking during pregnancy, Yes/No n/n	2/23	30/156	9/63	21/93
Smoker, Yes/No n/n	3/22	30/156	8/64	22/92
Other atopic disorders, yes	25 (100%)^b^	72 (39%)^a^	72 (100%)	-
Allergic rhinitis, yes	23 (92%)^b,c^	47 (25%)^a^	47 (65%)^a^	-
Atopic dermatitis, yes	8 (32%)^b^	12 (6%)^a^	12 (17%)	-
Allergic conjunctivitis, yes	17 (68%)^b^	26 (14%)^a^	26 (36%)	-
Food allergy, yes	5 (20%)^b^	10 (5%)^a^	10 (14%)	-
Drug allergy, yes	5 (20%)	14 (8%)	14 (19%)	-
Parental asthma, yes	6 (24%)^b,d^	11 (6%)^a^	6 (8%)	5 (4%)^a^
Parental allergic rhinitis, yes	10 (40%)^b,d^	36 (19%)^a^	21 (29%)^d^	15 (13%)^a,^^c^
Parental atopic dermatitis, yes	3 (12%)	8 (4%)	2 (3%)	6 (5%)
Antibiotics consumption, yes	11 (44%)	57 (31%)	22 (31%)	35 (31%)
Paracetamol consumption, yes	18 (72%)	107 (56%)	39 (54%)	68 (60%)
Inhaled corticosteroids consumption, yes	2 (8%)	5 (3%)	3 (4%)	2 (2%)
Injectable corticosteroids consumption, yes	2 (8%)	7 (4%)	6 (8%)^d^	1 (1%)^c^
Blood leucocyte count /µl, mean (range)	9548 (6380–16,610)	9247 (5270–16,720)	9054 (5270–15,560)	9369 (5270–16,720)
Blood monocyte count /µl, mean (range)	590 (320–1080)	614 (240–1460)	618 (370–1160)	611 (240–1460)
Blood lymphocyte count /µl, mean (range)	1847 (1160–3130)	2017 (770–4290)	2021 (960–3600)	2015 (770–4290)
Blood basophil count /µl, mean (range)	32 (10–60)	31 (0–100)	28 (10–60)	32 (0–100)
Blood eosinophil count /µl, mean (range)	213 (70–350)^b,c,d^	138 (0–500)^a^	131 (0–380)^a^	143 (0–500)^a^
Blood neutrophil count /µl, mean (range)	6866 (3960–12,780)	6447 (3380–11,360)	6255 (3380–11,360)	6568 (3380–10,970)
**Season at sampling**				
Winter/spring/summer/autumn %	24.0/20.0/20.0/36.0	25.3/10.8/22.0/41.9	38.9/11.1/19.4/30.6 ^d^	16.7/10.5/23.7/49.1 ^c^

*A-AD* asthmatics with other coexisting atopic diseases; *NA* non-asthmatics; *NA-NAD* non-asthmatics without atopic diseases; *NA-AD* non-asthmatics with other atopic diseases.

^a^Significantly different (p-value < 0.05) from asthmatics with coexisting other atopic diseases (A-AD).

^b^Significantly different (p-value < 0.05) from non-asthmatics (NA).

^c^Significantly different (p-value < 0.05) from non-asthmatics with other atopic diseases (NA-AD).

^d^Significantly different (p-value < 0.05) from non-asthmatic without atopic diseases (NA-NAD).

**Table 2:** Characteristics of the study population: women of childbearing age (Validation set—Group 2).

	Group 2 (n = 126)^†^			
	A-AD	NA	NA-AD	NA-NAD
Subjects n	14	111	47	64
Age (years) mean (range)	33.0 (25–39)	33.3 (18–42)	33.0 (18–42)	33.5 (22–41)
BMI (kg/m^2^) before pregnant mean (range)	23.50 (18.46–34.37)	23.28 (15.94–39.91)	23.35 (17.63–37.29)	23.23 (15.94–39.91)
**Social class**				
Managers-technicians/skilled/semiskilled-unskilled/unemployed %	28.6/50.0/14.3/7.1	39.6/20.7/18.0/21.6	29.8/19.1/23.4/27.7	46.9/21.9/14.1/17.2
**Educational level**				
Incomplete secondary or less/complete secondary/university %	7.1/35.7/57.1	11.7/28.8/59.5	14.9/31.9/53.2	9.4/26.6/64.1
Smoking during pregnancy, Yes/No n/n	2/12	14/97	4/43	10/54
Smoker, Yes/No n/n	3/11	13/98	6/41	7/57
Other atopic disorders, yes	14 (100%)^b^	47 (42%)^a^	47 (100%)	–
Allergic rhinitis, yes	14 (100%)^b,c^	34 (31%)^a^	34 (72%)^a^	–
Atopic dermatitis, yes	5 (36%)^b^	13 (12%)^a^	13 (28%)	–
Allergic conjunctivitis, yes	8 (57%)^b^	20 (18%)^a^	20 (43%)	–
Food allergy, yes	1 (7%)	7 (6%)	7 (15%)	–
Drug allergy, yes	3 (21%)	8 (7%)	8 (17%)	–
Parental asthma, yes	3 (21%)	12 (11%)	5 (11%)	7 (11%)
Parental allergic rhinitis, yes	7 (50%)	34 (31%)	19 (40%)	15 (23%)
Parental atopic dermatitis, yes	2 (14%)	11 (10%)	6 (13%)	5 (8%)
Antibiotics consumption, yes	1 (7%)	32 (29%)	12 (26%)	20 (31%)
Paracetamol consumption, yes	8 (57%)	67 (60%)	31 (66%)	36 (56%)
Inhaled corticosteroids consumption, yes	2 (14%)^b,d^	1 (1%)^a^	1 (2%)	0 (0%)^a^
Injectable corticosteroids consumption, yes	0 (0%)	2 (2%)	1 (2%)	1 (2%)
Blood leucocyte count /µl, mean (range)	8537 (7050–11,570)	9268 (5180–15,310)	9258 (5760–14,090)	9276 (5180–15,310)
Blood monocyte count /µl, mean (range)	630 (450–960)	595 (320–1280)	601 (360–1030)	590 (320–1280)
Blood lymphocyte count /µl, mean (range)	2035 (1270–2650)	2002 (990–3790)	1988 (1110–3790)	2013 (990–3700)
Blood basophil count /µl, mean (range)	34 (20–60)	34 (0–90)	37 (10–80)	33 (0–90)
Blood eosinophil count /µl, mean (range)	234 (90–510)^b,c,d^	146 (20–430)^a^	159 (20–430)^a^	135 (30–430) ^a^
Blood neutrophil count /µl, mean (range)	5604 (4390–8320)	6491 (3470–10,990)	6473 (3510–9790)	6504 (3470–10,990)
**Season at sampling**				
Winter/spring/summer/autumn %	0.0/35.7/35.7/28.6	3.6/32.4/41.4/22.5	2.1/36.2/38.3/23.4	4.7/29.7/43.8/21.9

*A-AD* asthmatics with other coexisting atopic diseases; *NA* non-asthmatics; *NA-NAD* non-asthmatics without atopic diseases; *NA-AD* non-asthmatics with other atopic diseases.

^a^Significantly different (p-value < 0.05) from asthmatics with coexisting other atopic diseases (A-AD).

^b^Significantly different (p-value < 0.05) from non-asthmatics (NA).

^c^Significantly different (p-value < 0.05) from non-asthmatics with other atopic diseases (NA-AD).

^d^Significantly different (p-value < 0.05) from non-asthmatic without atopic diseases (NA-NAD).

^†^One woman was not included in any of the four categories for being asthmatic without other atopic diseases.

### Predictive modeling for VOC discriminant identification in exhaled breath analysis

No grouping by asthma disease was observed with exploratory data analysis based on principal component analysis (PCA) (Supplemental Fig. S3). However, grouping by seasonal variation in sampling was observed for all sample types: exhaled breath samples and ambient air samples (Supplemental Figs. S4, S5). Therefore, ANOVA (analysis of variance)-simultaneous component analysis (ASCA) was performed to avoid seasonal variation influences. The factors selected were the season of measurement and the usual residence zone defined by air quality modeling using the Weather Research and Forescasting (WRF) + CHIMERE modeling system, considering levels of ozone (O_3_), nitrogen dioxide (NO_2_), sulfur dioxide (SO_2_) and particulate matter^34,35^.

**Figure 5:**
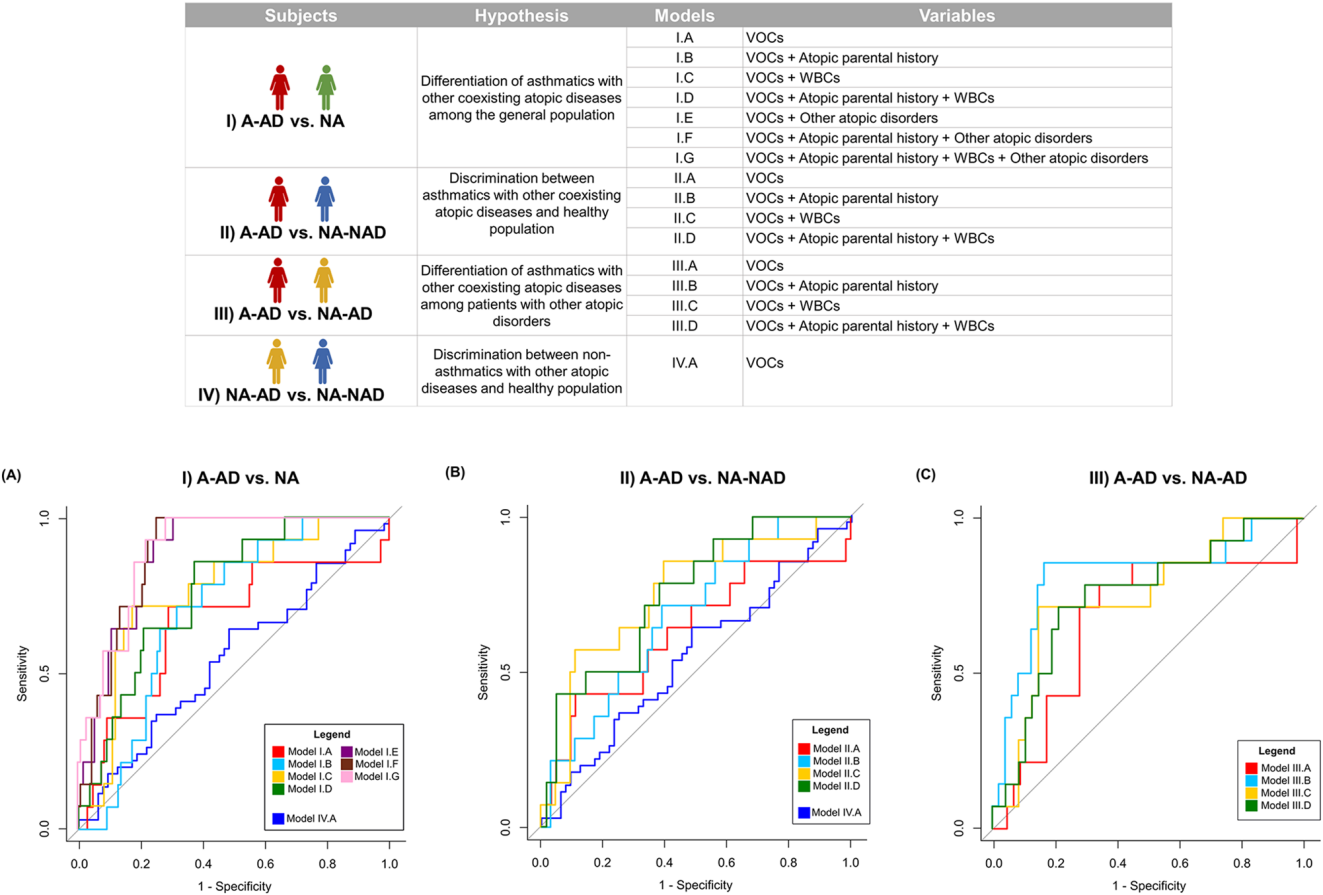
The constructed models. Top panel. Characteristics of the constructed models. (WBCs: white blood cell counts). Bottom panel. Receiver operator characteristic (ROC) curves of the constructed models. (**A**) ROC curves for discriminating between A-AD (asthmatics with other coexisting atopic diseases) and NA (non-asthmatics). (**B**) ROC curves for discriminating between A-AD and NA-NAD (non-asthmatic without atopic diseases). (**C**) ROC curves for discriminating between A-AD and NA-AD (non-asthmatic with other atopic diseases).

The properties and receiver operator characteristic (ROC) curves of the constructed models are shown in Fig. 5. For more information, the formulas of the models are provided in Supplemental Table S2, and the attributes of the features selected by the models are detailed in Supplemental Table S3. The compound including feature F62 had an ion fragmentation pattern characteristic of several compounds of the tetrahydroisoquinoline family; therefore, this compound was named a “tetrahydroisoquinoline derivative” and not with a specific name. Compound identification of all selected features was conducted based on mass spectra and retention times matching with the NIST (National Institute of Standard and Technology) library and commercial standards (match factor and error RI (retention index error) value computation), except for three compounds (isoprene, tetrahydroisoquinoline derivative and 2-propenoic acid, 3-(2-hydroxyphenyl)-), which were identified based only on mass spectral library matching. The extent to which the prediction accuracy of the constructed models and variables were used in each model are summarized in Table 3.

**Table 3:** Accuracy of constructed models and selected variables in each model (discriminant volatile organic compounds (VOCs) and other variables).

Models	AUC_CV_	p-value	AUC_VS_	Sensitivity (%)	Specificity (%)	VOCs	Other variables
**(I) A-AD vs NA**							
I.A	0.70	**0.017**	0.67	71	63	Acetone (CAS number: 67-64-1) Tetrahydroisoquinoline derivative 2-Ethyl-1-hexanol (CAS number: 104-76-7)	
I.B	0.73	**0.003**	0.70	71	60	Acetone (CAS number: 67-64-1) Tetrahydroisoquinoline derivative 2-Ethyl-1-hexanol (CAS number: 104-76-7)	Parental asthma Parental rhinitis Parental dermatitis
I.C	0.79	**0.001**	0.77	71	65	Tetrahydroisoquinoline derivative 2-Ethyl-1-hexanol (CAS number: 104-76-7)	Blood eosinophil count
I.D	0.80	**0.001**	0.76	86	63	Tetrahydroisoquinoline derivative 2-Ethyl-1-hexanol (CAS number: 104-76-7)	Parental asthma Parental rhinitis Blood eosinophil count
I.E	0.88	**0.001**	0.88	100	69	Carbon disulfide (CAS number: 75-15-0) Tetrahydroisoquinoline derivative 2-Ethyl-1-hexanol (CAS number: 104-76-7)	Allergic rhinitis Atopic dermatitis Allergic conjunctivitis Food allergy Drug allergy
I.F	0.90	**0.001**	0.89	100	70	Acetone (CAS number: 67-64-1) Carbon disulfide (CAS number: 75-15-0) Tetrahydroisoquinoline derivative 2-Ethyl-1-hexanol (CAS number: 104-76-7)	Parental asthma Parental dermatitis Allergic rhinitis Atopic dermatitis Allergic conjunctivitis Food allergy Drug allergy
I.G	0.91	**0.001**	0.90	100	68	Carbon disulfide (CAS number: 75-15-0) Tetrahydroisoquinoline derivative 2-Ethyl-1-hexanol (CAS number: 104-76-7)	Parental asthma Parental dermatitis Blood eosinophil count Allergic rhinitis Atopic dermatitis Allergic conjunctivitis Food allergy Drug allergy
**(II) A-AD vs NA-NAD**							
II.A	0.68	**0.031**	0.63	64	55	Acetone (CAS number: 67-64-1) Tetrahydroisoquinoline derivative 2-Ethyl-1-hexanol (CAS number: 104-76-7)	
II.B	0.73	**0.008**	0.68	71	58	Acetone (CAS number: 67-64-1) Tetrahydroisoquinoline derivative 2-Ethyl-1-hexanol (CAS number: 104-76-7)	Parental asthma Parental rhinitis
II.C	0.77	**0.001**	0.75	64	68	Acetone (CAS number: 67-64-1) Tetrahydroisoquinoline derivative 2-Ethyl-1-hexanol (CAS number: 104-76-7)	Blood eosinophil count
II.D	0.79	**0.002**	0.75	71	62	Acetone (CAS number: 67-64-1) Tetrahydroisoquinoline derivative 2-Ethyl-1-hexanol (CAS number: 104-76-7)	Parental asthma Parental rhinitis Blood eosinophil count
**(III) A-AD vs NA-AD**							
III.A	0.70	**0.025**	0.68	71	72	Carbon disulfide (CAS number: 75-15-0) Tetrahydroisoquinoline derivative Decane (CAS number: 124-18-5) 2-Ethyl-1-hexanol (CAS number: 104-76-7) 2,2,4-Trimethyl-1,3-pentanediol diisobutyrate (TXIB) (CAS number: 6846-50-0)	
III.B	0.67	**0.042**	0.81	86	60	Carbon disulfide (CAS number: 75-15-0) Tetrahydroisoquinoline derivative Decane (CAS number: 124-18-5) 2-Ethyl-1-hexanol (CAS number: 104-76-7) 2,2,4-Trimethyl-1,3-pentanediol diisobutyrate (TXIB) (CAS number: 6846-50-0)	Parental asthma Parental dermatitis
III.C	0.80	**0.001**	0.74	71	60	Carbon disulfide (CAS number: 75-15-0) Tetrahydroisoquinoline derivative Decane (CAS number: 124-18-5) 2-Ethyl-1-hexanol (CAS number: 104-76-7) 1,2-Benzenedicarboxylic acid, bis(2-methylpropyl) ester (CAS number: 84-69-5)	Blood eosinophil count Blood lymphocyte count
III.D	0.82	**0.001**	0.75	79	70	Carbon disulfide (CAS number: 75-15-0) Tetrahydroisoquinoline derivative Decane (CAS number: 124-18-5) 2-Ethyl-1-hexanol (CAS number: 104-76-7) 1,2-Benzenedicarboxylic acid, bis(2-methylpropyl) ester (CAS number: 84-69-5)	Parental asthma Parental dermatitis Blood eosinophil count Blood lymphocyte count
**(IV) NA-AD vs NA-NAD**							
IV.A	0.55	0.236	0.54	46	62	Isoprene (CAS number: 78-79-5) 2-Propenoic acid, 3-(2-hydroxyphenyl)-, (E)-(CAS number: 614-60-8) 2,2,4-Trimethyl-1,3-pentanediol diisobutyrate (TXIB) (CAS number: 6846-50-0)	

*A-AD* asthmatics with other coexisting atopic diseases; *NA* non-asthmatics; *NA-NAD* non-asthmatics without atopic diseases; *NA-AD* non-asthmatics with other atopic diseases; *AUC* area under the receiver operating characteristic curve; *AUCcv* AUC obtained by fivefold cross-validation; *AUCvs* AUC obtained by testing in validation set.

#### Asthmatics with other coexisting atopic diseases (A-AD) vs. Non-asthmatics (NA)

Seven models (model I.A, model I.B, model I.C, model I.D, model I.E, model I.F and model I.G) were generated to distinguish between asthmatics with other coexisting atopic diseases and non-asthmatics. All models were significant (p-value < 0.05 in permutation test), and selected features of 2-ethyl-1-hexanol and tetrahydroisoquinoline derivative acted as discriminants in all models. The features of acetone, 2-ethyl-1-hexanol and a tetrahydroisoquinoline derivate were selected in model I.A, which included only VOC measurements. The AUC (area under the receiver operating characteristic curve) value of this model in the validation set was 0.67 (71% sensitivity and 63% specificity). Model I.B, which also included atopic parental history, selected the same features set as model I.A as well as variables related to atopic antecedents (parental asthma, parental rhinitis and parental dermatitis).

#### Asthmatics with other coexisting atopic diseases (A-AD) vs. Non-asthmatics without atopic diseases (NA-NAD)

Four models were constructed to distinguish between asthmatics with other coexisting atopic diseases and non-asthmatics without atopic diseases. The features of acetone, 2-ethyl-1-hexanol and a tetrahydroisoquinoline derivative were selected in the four models, and all models were significant (p-value < 0.05 in permutation test).

#### Asthmatics with other coexisting atopic diseases (A-AD) vs. Non-asthmatics with other atopic diseases (NA-AD)

Four models were constructed (model III.A, model III.B, model III.C and model III.D)) for asthma discrimination among women who suffer any atopic disease. All models showed statistical significance (p-value < 0.05 in permutation tests). The features of carbon disulfide, a tetrahydroisoquinoline derivative, decane and 2-ethyl-1-hexanol were selected in the four models.

#### Non-asthmatics with other atopic diseases (NA-AD) vs. Non-asthmatics without atopic diseases (NA-NAD)

Model IV.A was unable to discriminate between non-asthmatics with other atopic diseases and non-asthmatics without atopic diseases. Moreover, it was not significant (p-value = 0.236), and the accuracy in the validation set was poor (AUC = 0.54, 46% sensitivity and 62% specificity).

The levels in exhaled breath of the features selected by the fifteen significant models were not influenced by either smoking habits or consumption of drugs reported by women (antibiotics, paracetamol, inhaled corticosteroids and injectable corticosteroids), except for the levels of 2,2,4-Trimethyl-1,3-pentanediol diisobutyrate (99 m/z) which were significantly higher in women who consumed paracetamol (p-value = 0.013) (Supplementary Figs. S6–S10). However, no significant differences in paracetamol consumption were observed between the four categories (A-AD, NA, NA-AD and NA-NAD) (Table 1 and Table 2). Furthermore, intensities of features from discriminant VOCs were significantly higher in human exhaled breath samples than in ambient samples (Supplementary Fig. S11). Moreover, 2,2,4-Trimethyl-1,3-pentanediol diisobutyrate (99 m/z) was not detected in environmental samples.

## Discussion

Results of the two last health survey in Spain (EHSS-2014 and ENSE-2017) elaborated by INE (the National Institute of Statistics of Spain) data analysis indicated a gender bias in asthma disease in Spain, owing to its high prevalence in women. In plots of MCA results of both surveys (Fig. 2A and Supplemental Fig. S1), age of subjects was the variable with the highest contribution to Dimension 1. Category "woman" of gender variable was extremely close to the category "asthma_Yes" in both dimensions (Dimension 1 and Dimension 2). In addition, asthma disease was very close in Dimension 2 to other diseases such as thyroid problems, other atopic disorders (any atopic disease excluding asthma) or skin disorders. Moreover, a significant relationship was observed between asthma and other diseases such as skin disorders, other atopic disorders, thyroid problems, diabetes, kidney problems, arterial hypertension or osteoporosis (Supplemental Table S1). Asthma influences some chronic diseases such as coronary heart disease, diabetes mellitus, and hypertension, but the impact on vital diseases such chronic kidney disease is not yet verified. However, patients with bronchial asthma may have increased risk of developing chronic kidney disease^36^. The connection between asthma and thyroid problems has been noted^37,38^ but the effects of thyroid hormones on airway contractility are unclear. Prevalence of thyroid problems is higher in women. It is reported that the sex bias could be due to the fact that hormone regulation may play a relevant role in thyroid problems^10,39^. Nevertheless, no significant association between asthma and thyroid problems, diabetes, kidney problems and osteoporosis were observed when only data from 18- to 45-year-old women were selected in both health surveys (Supplemental Table S1). On the other hand, results of data analysis of open cohorts of 18- to 45-year-old women confirmed that asthma disease is associated to skin disorder and other atopic disorders. The category “asthma_Yes” was very close to categories “skin_disorders_Yes” and “other atopic disorders_Yes” in Dimension 1 in both plots of MCA (Fig. 2B and Supplemental Fig. S1). As a result, the factors most associated with asthma disease in adults are gender, other atopic disorders and skin disorders. Thus, this paper shows the first study focused on discrimination of asthma with other coexisting atopic diseases in women of childbearing age using a noninvasive technique such as VOC analysis in exhaled breath.

Although VOC analysis of exhaled air has been proposed as a potential strategy for the diagnosis and monitoring of asthma, its implementation in clinical practice has been impossible so far. In this sense, metabolomics practice is crucial to overcome the current limitations to its integration into day-to-day clinical practice^29,33^. Thus, a metabolomics perspective was used in this study. So, a large cohort of subjects were recruited, and a room air content sample was collected for each participant sample to assess possible contamination through the sample collection and analysis processes. Special emphasis has been placed on the data preprocessing step, which is a classic bottleneck in exhaled breath analysis by GC/MS. The biggest challenge for the development of the technique has been to address the preprocessing of the data to transform it into a useful matrix for data analysis. In fact, a reproducible and transparent workflow developed by our group for data preprocessing using open sources has been implemented for the first time for biomarker discovery in the present study^40^. VOC identification was performed based on spectral similarity and retention times. Once this phase is over, robust data analysis with adequate validation are required to measure the predictive performance of a statistical model with reliable predictions of unseen cases.

In this sense, a novel combination of robust techniques (ASCA and LASSO + LR (least absolute shrinkage and selection operator + logistic regression)) was applied for data analysis. Although these techniques have previously been used independently for VOC analysis of exhaled air, since ASCA was used by van de Kant et al.^41^ and LASSO + LR by Monasta et al.^42^, both statistical tests have to our knowledge never been implemented together in hypothesis testing. ASCA is a useful tool for large longitudinal cohorts, as many factors (e.g., season of measurement) can indirectly influence the results^43^. On the other hand, LASSO + LR allows for high-dimensional data analysis without being a "black box", as are other supervised learning techniques, such as support vector machines (SVMs). In fact, it is as easy to interpret as conventional logistic regression, which is essential in the medical field. Another advantage of LASSO is its ability to select variables and identify discriminant features^44^. Here, the models constructed were validated by cross-validation and testing using an independent set that was not involved in the model-building process. To date, few studies carry out this statistical treatment. This is of course computationally expensive, but worth it as the bias introduced by improper performance estimation can be large. In addition, this is the first study about exhaled breath analysis using GC/MS focused in asthma disease where the statistical significance of each model was assessed by permutation test (p-value was computed)^29,33^. Nevertheless, model validation in another set of subjects outside the NELA cohort would be of interest in the future.

This study has shown that discrimination between asthmatics with other coexisting atopic diseases and non-asthmatics (with or without atopic diseases) using exhaled breath analysis is feasible when metabolomics best practices are implemented. All constructed models that included subjects with asthma were significant (p-value < 0.05 in permutation tests). In addition, overfitting of the models was low, since the performance was similar for both cross-validation and testing using the validation set. This ensures good generalization performance of the model to new unknown samples. Furthermore, although the accuracy of the models which also involved other variables related to atopic parental history and white blood cell counts (WBCs) were higher than the models that included only VOC variables, the same features were selected. In fact, features from acetone, tetrahydroisoquinoline derivative and 2-ethyl-1-hexanol were selected as discriminants of asthma with other coexisting atopic diseases. Accordingly, process reliability modeling confirmed that VOC analysis together with atopic parental history is sensitive for discriminating asthma with other coexisting atopic diseases.

Acetone is a secondary product of lipid peroxidation of polyunsaturated fatty acids (PUFAs)^45,46^, and a positive association between asthma and acetone levels in exhaled breath has been previously reported^47^. 2-ethyl-1-hexanol is an indoor contaminant, because it is the main metabolite of di(2-ehylhexyl)phthalate, which is a frequent plasticizer of polyvinylchloride (PVC)^48–50^. Moreover, levels of this compound rise with increased relative humidity rising in homes^51^. However, the intensity of 2-ethyl-1-hexanol in the Tedlar bags was found to be negligible compared to the exhaled breath samples (Supplementary Fig. S12). Furthermore, besides being an indoor contaminant, it is also well documented that 2-ethyl-1-hexanol is an endocrine-disrupting chemical (EDC)^52–54^. Evidence supports that EDCs may be associated with increased oxidative stress and modulate the immunological response^55^. Previous studies indicate that exposure to 2-ethyl-1-hexanol increases CD4 + T cell activation and asthma prevalence^52,56^. Moreover, a significant increase in 2-ethyl-1-hexanol has been observed from lung cancer in exhaled breath^57^, as well as, in cancer cell lines of different histological origins^58^. In addition, ethyl-1-hexanol is considered an exogenous substance that induces the proliferation of peroxisomes in the liver^59^. On the other hand, the tetrahydroisoquinoline derivative containing 5-phenyl-2-furan exhibits considerable inhibitory activity of PD4 phosphodiesterases, increasing the intracellular concentration of the secondary signal messenger cyclic adenosine monophosphate (cAMP)^60^. These compounds have been extensively studied as anti-inflammatory drugs^60^. The tetrahydroisoquinoline skeleton is commonly found in pharmaceutical drugs, notably quaternary ammonium muscle relaxants. Tetrahydroisoquinoline derivatives may be formed in the body as metabolites of some drugs; they are usually located in the cell membrane, and their neurotoxicity, among other aspects, depends on their propensity to form free radicals^61^. Endogenous production of neurotoxic tetrahydroisoquinoline derivatives such as norsalsolinol continues to be investigated as possible causes for some conditions, such as Parkinson's disease^62^. Nevertheless, no significant relationship was observed between drugs reported by the women included in our study and levels of the tetrahydroisoquinoline derivative (Supplementary Figs. S7–S10). On the other hand, although previous studies show that several compounds such as hydroquinones can be Tenax degradation products^63^, the intensity observed of the tetrahydroisoquinoline derivative in the reconditioned Tenax tubes was marginal compared to exhaled breath samples (Supplementary Fig. S12).

Most volatilome studies focus exclusively on the comparison of those with asthma and healthy controls. However, it is also important to compare the exhaled breath of patients with asthma and those with similar symptoms^33^. Moreover, since the most asthmatics have other coexisting diseases^6,23^, it is crucial to take them into account for the correct study of this disease. So, in this study, exhaled breath profiles of A-AD and NA-AD were compared, successfully discriminating between the two categories (four significant models were generated). In this regard, Dragonieri et al*.*^64^ distinguished between asthma patients with allergic rhinitis and patients without asthma but with allergic rhinitis. However, they could not determine the identity of discriminant VOCs, and only a “breathprint” was obtained because e-NOSE instead of technologies based on mass spectrometry was used. In our study, VOCs with features selected as discriminants of asthma disease among patients with other atopic disorders were carbon disulfide, tetrahydroisoquinoline derivative, 2-ethyl-1-hexanol and decane in model III.A, model III.B, model III.C and model III.D. Carbon disulfide is an environmental pollutant classified as neurotoxic that has been previously selected as a discriminant for asthma^65^. On the other hand, the endogenous origin of alkanes in exhaled breath is under debate, as they can also derive from exogenous sources^66^. Regardless, decane has been highlighted as a discriminant of allergic asthma by previous studies^67,68^. Traditionally, alkanes have been identified as a possible biomarker for asthma diagnosis^69^ because some of them are produced during lipid peroxidation^24,70,71^ (Fig. 1).

In this study, in addition to the main VOC variables, other variables in addition to VOC variables were included in the model-building process, such as atopic parental history or WBCs, with successful outcomes. This is in line with the results of previous studies which have also shown that analysis of VOCs in exhaled breath can be compatible with established strategies^72–74^. In this regard, a radical change of concept in asthma diagnosis is urgently being demanded by physicians and scientists. Hence, the diagnostic protocol must be based on a combination of techniques that are preferably noninvasive and do not rely on a single method.

### Study limitations

The study has several limitations. It was not possible to distinguish between asthma phenotypes. All but one of the asthmatics included in the study were diagnosed with other atopic diseases. Therefore, it was not possible to differentiate between asthmatics with coexisting other atopic diseases and asthmatics without other atopic diseases. Furthermore, other pharmacological treatments not included in the questionnaires may have a confounding effect that could affect the exhaled breath samples.

## Conclusion

The results of the present study mainly show that the distinction between asthmatics with other coexisting atopic diseases and non-asthmatics (with or without atopic diseases) using VOC analysis in exhaled breath is feasible when metabolomics best practices are performed (e.g., a large cohort of subjects were recruited, environmental influence was assessed, a reproducible workflow was used by data preprocessing step, a robust data analysis was carried out for models construction, the model performance was assessed by two approaches (fivefold cross-validation and testing in the validation set), and significance of models was evaluated). In addition, the output of the modeling process confirms that VOC analysis together with the subject's parental history can be a good strategy for asthma with other coexisting atopic diseases screening. Moreover, the findings of this study confirmed that VOC analysis (either by itself or together with other established techniques) is helpful to distinguish asthma among patients with other atopic diseases.

## Methods

### Analysis of the data collected in the European Health Survey in Spain of 2014 (EHSS-2014) and in the Spanish National Health Survey of 2017 (ENSE-2017)

The information compiled in both the European Health Survey in Spain 2014 (EHSS-2014) and the Spanish National Health Survey 2017 (ENSE-2017), elaborated by the National Institute of Statistics (INE) in Spain was analysed using R (version 3.6.1). Specifically, a multiple correspondence analysis (MCA) was performed using package *FactoMineR*^75^. In addition, chi-square test or Fisher's exact test was carried out to check if there were significant differences (p-value < 0.05) in variables between asthmatics and non-asthmatics^76^. Firstly, the analysis was conducted on data from all subjects and, secondly, only on data from women of childbearing age (18- to 45-year-old women).

### Study design and participants

The data used comes from subjects of the Nutrition in Early Life and Asthma (NELA) study (www.nela.imib.es), a prospective population-based birth cohort set up in Murcia (Spain)^34,35,40^. The study protocol was reviewed and approved by the Ethics Committee of the Virgen de la Arrixaca Clinical University Hospital (HCUVA) in accordance with the guidelines of The Declaration of Helsinki. Written informed consent was obtained from participants at recruitment.

Recruitment of pregnant women was carried out during 36 months (March 2015–April 2018) at the time of ultrasound control at 20 weeks of gestation at the Maternal–Fetal Unit at HCUVA. The enrolled subjects had several follow-up points: follow-up visit 1 (at 20–24 weeks of pregnancy), follow-up visit 2 (at 32 weeks of pregnancy), follow-up visit 3 (at delivery), follow-up visit 4 (3 months after childbirth) (Supplementary Fig. S13). The inclusion criteria included: usual residence in Health Area I and certain districts of Health Areas VI and VII of the Region of Murcia; planning to live in the area of study for at least 2 years; intention to give birth at the reference hospital; Spanish Caucasian origin; 18–45 years of age; singleton pregnancy; nonassisted conception; and normal echography at 20 weeks of gestation (no major malformations). The exclusion criteria included: existing chronic disease; pregnancy complications (except gestational diabetes and hypertensive disorders); and not intending to deliver in the reference hospital.

Among the 1350 women invited to participate, 738 were ultimately enrolled in the NELA study. Exhaled breath sampling was conducted at follow-up visit 4 (3 months after childbirth) between May 2017 and October 2018. During that period, it was collected exhaled breath from 337 women who were included in the present study (Supplementary Fig. S13).

### Information on asthma and other atopic disorders

Information for women of reproductive age with a medical history of asthma and other atopic manifestations was collected through a structured questionnaire administered in person by trained interviewers at 20–24 weeks of gestation (follow-up visit 1). Women who reported asthma were defined as having a positive response to the question *‘Have you ever been diagnosed with asthma?*’. Women who reported other atopic disorders were defined as having a positive response to the question *“Have you ever been diagnosed with allergic rhinitis, atopic dermatitis, allergic conjunctivitis, food allergy and drug allergy?*”. Thus, the women were divided into four categories: asthmatics with other coexisting atopic diseases (A-AD), non-asthmatics with other atopic diseases (NA-AD), non-asthmatics without atopic diseases (NA-NAD), and non-asthmatics (NA) (this category includes both NA-AD and NA-NAD) (Fig. 3). Information on parental history of asthma (yes/no), allergic rhinitis (yes/no) and atopic dermatitis (yes/no) was also collected by questionnaire.

### Other variables

Using questionnaires administered in person during pregnancy, we obtained information through about the following: age; social class (defined as occupation during pregnancy based on the highest social class by using a widely used Spanish adaptation of the international ISCO88 coding system: I–II, managers/technicians; III, skilled; IV–V, semiskilled/unskilled; and unemployed)^77^; educational level (incomplete secondary or less, complete secondary, and university); smoking during pregnancy (yes/no); antibiotics consumption (yes/no); paracetamol consumption (yes, no), inhaled corticosteroids consumption (yes/no); and injectable corticosteroids consumption (yes/no). Prepregnancy body mass index (BMI) based on height and prepregnancy self-reported weight (kg/m^2^^2^) were calculated. White blood cell counts (WBCs) in blood samples of the pregnant women were determined using a Sysmex® XN9000 (Sysmex Corporation, Kobe, Japan) hematology analyzer that combines light scatter and optical detection with electrical impedance. In addition, information about smoking habits (smoker (yes/no)) at the sampling of exhaled breath (follow-up visit 4) was obtained by questionnaires administered in person three months post gestation. The season at the sampling of exhaled breath (winter, December-February; spring, March–May; summer, June–August; and autumn, September–November) was also considered.

### Breath sampling

Breath sampling was performed at 3 months after childbirth (follow-up visit 4) following the protocol described by Sola Martínez et al*.*^40^. Briefly, exhaled breath was collected in 1 L Tedlar gas sampling bags. Specifically, mixed breath sample (alveolar and dead space) were collected. Then, the exhaled breath contained in the gas sampling bags was immediately transferred to to thermal desorption tubes (Tenax TA, Markes International) to avoid the diffusion through the bag wall. A room air content sample was also collected directly through Tenax tube for each exhaled breath sample using an Easy-VOC syringe (Markes International) to control for environmental conditions at sampling. Almost all samples were analysed on the same day of collection, being a maximum storage period of less than one week. The Tenax tubes were stored at 4 °C for storage periods longer than one day. Tenax tubes were heated to 335ºC for 25 min for reconditioning after each use. The Tedlar bags were cleaned with 10 nitrogen flushes (99.9% nitrogen purity) before use. Thus, the levels of background artefacts (N,N-dimethylacetamide and phenol) in the gas sampling bags were strongly reduced (Supplementary Fig. S12).

### Exhaled breath analysis and data preprocessing

Exhaled breath analysis and data preprocessing were performed using a protocol previously detailed^40^. The breath samples were analyzed using a thermal desorption system coupled with gas chromatography-single quadrupole mass spectrometry (TD-GC/q-MS). In GC/MS analysis, hundreds of features (ion peaks with a retention time and a characteristic m/z signal) were obtained from the fragmentation of compounds of the exhaled breath in the mass spectra^78^. Then, the raw data were converted to mzXML. format by *MsConvert* from *Proteowizard*^79,80^. Later, an open source workflow that used the functions of three packages (*xcms*^81^, *cliqueMS*^82^ and *eRah*^78^)written in the R language was conducted for data preprocessing. This workflow enables integration between the two main approaches for data preprocessing from GC/MS analysis (feature detection and compound detection). Thus, a matrix of relative intensities of features from breath samples was obtained. In addition, it was able to determine which detected feature belongs to each chemical compound. Furthermore, compound identification was carried out by matching with the NIST (National Institute of Standard and Technology) spectral library and by calculating two factors (match factor and retention index error) using the *eRah*^78^ package. For retention index error computation, retention times of two commercial standards (C7-C30 saturated alkane standard and VOC calibration standard, Sigma-Aldrich) and retention indexes of the compounds recorded in the NIST library were used.

### Data analysis

#### Subject characteristics. Associations between atopic conditions

The study subjects (n = 336) were randomly divided into two sets according to the sampling date: Group 1 (n = 211) and Group 2 (n = 125). Exhaled breath samples collected between May 2017 and February 2018 constituted Group 1, and exhaled breath samples collected between March 2018 and October 2018 constituted Group 2.

The characteristics of the study population were analyzed by R version 3.6.1 to identify differences between asthmatic and non-asthmatic cases and associations with other atopic conditions. Shapiro–Wilk and Lilliefors tests (*nortest*^83^ package) were employed to assess a normal distribution of the data. According to the data distribution, parametric tests (Student's t-test or ANOVA test) or nonparametric tests (Mann–Whitney U test or Kruskal–Wallis test) were performed to confirm statistically significant differences between continuous variables (p-value < 0.05). Moreover, the chi-square test or Fisher's exact test was applied to assess differences between categorical variables.

#### Predictive modeling for VOC discriminant identification in exhaled breath analysis

**Figure 6:**
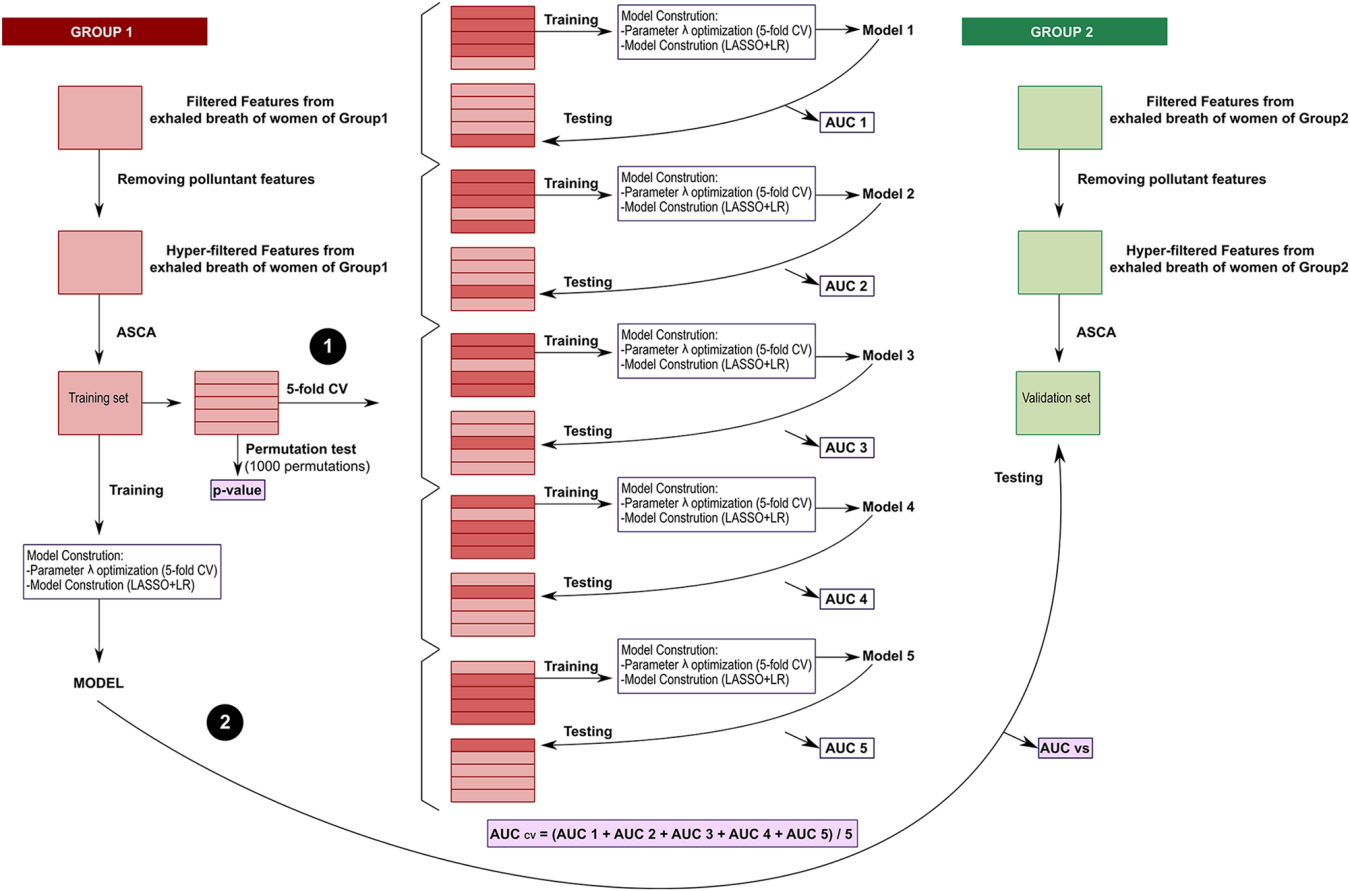
Data modeling workflow. The performance of the models was assessed by two approaches: (1) fivefold cross-validation and (2) testing in the validation set. *CV* cross-validation, *ASCA* ANOVA-simultaneous component analysis, *LASSO* least absolute shrinkage and selection operator, *LR* logistic regression, *AUC* area under the receiver operating characteristic curve, *AUC*_*cv*_ AUC obtained by fivefold cross-validation, *AUC*_*vs*_ AUC obtained by testing in the validation set.

The workflow carried out for data modeling using R version 3.6.1 is shown in Fig. 6. Matrices with relative intensities of filtered features obtained after data preprocessing were used for data analysis. Moreover, features due to contaminants of gas sampling bags (N,N-dimethylacetamide and phenol), pump oils and siloxanes from the GC/MS columns were discarded. To reduce the influence of exogenous pollutants in ambient air, nonnormalized intensities of the human sample features and room air content features were compared. For this purpose, Wilcoxon signed-rank tests were carried out to compare feature intensities between environmental samples and human exhaled breath samples of the NELA cohort; features with significantly higher intensities in room air content samples were also removed. The features resulting from this screening process were named hyper-filtered features.

Then, an initial exploratory analysis on the hyper-filtered features was performed using PCA (*FactoMineR*^75^ package) to detect trends, clusters and outliers. Afterward, ANOVA-simultaneous component analysis (ASCA) (*MetStaT*^84^ package) was conducted on hyper-filtered features to reduce possible variations induced by different experimental factors. This method allows separate contributions of different factors of variation in the original data matrix to obtain a residual matrix free of that influence (40).

In the modeling process, samples of Group 1 were used as a training set; samples of Group 2 were used as a validation set as shown in Fig. 6. Models were generated by least absolute shrinkage and selection operator (LASSO) and logistic regression (LR) using the R *glmnet*^85^ package. The process of model construction involves two steps: (1) parameter optimization and (2) model construction. LASSO + LR is a linear method based on a combination of a shrinkage method and a supervised learning technique. Although LASSO + LR is interpreted as a logistic regression that allows for analyzing the relationship between variables and calculating odds ratio values, the coefficient computation is different from that of conventional linear methods. Due to multicollinearity and high dimensionality issues, the coefficients of models obtained by conventional linear methods can reach extremely high values. LASSO incorporates a penalization in likelihood maximization through parameter λ during coefficient calculation. As many coefficients obtain a value of 0 after penalty, they are excluded. Therefore, avoiding or reducing overfitting is possible by variable selection^44,86^. Parameter λ was optimized by fivefold cross-validation.

The model performance was estimated through two approaches. First, the constructed model was validated by fivefold cross-validation. Samples of Group 1 were randomly divided into 5 subgroups. Four of them were used for training and the model-building process. Testing was conducted using the remaining subgroup, and ROC curves using the *pROC*^87^ package were determined to obtain AUCs. Cut-off values were computed automatically on the basis of the case/control balance in the training set of each model. This process was repeated 5 times for each model such that all subgroups were used as a testing set. The cross-validated AUC value, AUC_CV_, was calculated as the average of the AUC values of the 5 submodels. Moreover, permutation tests with 1000 permutations were carried out to obtain the statistical significance of the constructed models. It is important to note that in permutation tests, a p-value is obtained by comparing the performances of constructed models and predictive models with randomly permuted class labels. The second strategy involves performance evaluation of the constructed model on Group 2 exhaled samples (validation set) and obtaining the AUCvs by testing with the validation set.

Sixteen predictive models using the residual matrices were constructed, and their characteristics are shown in Fig. 5 (top panel). Apart from VOC variables, variables such as atopic parental history, white blood cell count or other atopic disorders were included in the corresponding models.

## Supplementary Information

Supplementary Information.
