# Supplementary material for: Exhaled volatilome analysis as a useful tool to discriminate asthma with other coexisting atopic diseases in women of childbearing age
Source: Sci Rep. 2021 Jul 5;11:13823. doi: 10.1038/s41598-021-92933-2 (PMC8257728; doi:10.1038/s41598-021-92933-2)
Supplement: Supplementary file 1 — Supplementary Information. [file 41598_2021_92933_MOESM1_ESM.pdf]

Exhaled volatilome analysis as a useful tool to discriminate asthma with other coexisting atopic diseases in women of childbearing age.

Rosa A. Sola-Martínez[1,2], Gema Lozano-Terol[1,2], Julia Gallego-Jara[1,2], Eva Morales[2,3], Esther Cantero-Cano[2], Manuel Sanchez-Solis[2,4,5,6] Luis García-Marcos[2,4,5,6], Pedro Jiménez-Guerrero[7], José Antonio Noguera-Velasco[1,2,8], Manuel Cánovas Díaz[1,2] and Teresa de Diego Puente[1,2]* on behalf of the NELA study group[2,#].

[1]Department of Biochemistry and Molecular Biology B and Immunology, University of Murcia, Spain.

[2]Biomedical Research Institute of Murcia, IMIB-Arrixaca, Murcia, Spain.

[3]Department of Public Health Sciences, University of Murcia, Spain.

[4]Respiratory and Allergy Units, Arrixaca Children's University Hospital, University of Murcia, Murcia, Spain.

[5]Department of Paediatrics, University of Murcia, Spain.

[6]Network of Asthma and Adverse and Allergy Reactions (ARADyAL), Health Institute Carlos III, Madrid, Spain.

[7]Regional Atmospheric Modelling Group, Department of Physics, University of Murcia, Murcia, Spain.

[8]Molecular Therapy and Biomarkers Research Group, Clinical Analysis Service, University Clinical Hospital "Virgen de la Arrixaca", University of Murcia, Spain.

[#]A full list of the NELA study group can be found in the Acknowledgements.

***Correspondence author:**

Teresa de Diego Puente

Associate Professor.

Biotechnology Group

Dept. of Biochemistry and Molecular Biology B and Immunology
Faculty of Chemistry. University of Murcia.
ResearcherID: N-9215-2013
Orcid ID: 0000-0003-3501-5483
https://scholar.google.com/citations?user=FrGSyt4AAAAJ&hl=en
Phone. +34 868 88 73 95
tdp@um.es

ACKNOWLEDGMENTS

This work was supported by grants from the Instituto de Salud Carlos III, Spanish Ministry of Science, Innovation and Universities, and Fondos FEDER (grant numbers CP14/00046, PIE15/00051, PI16/00422 and ARADyAL network RD160006), the Ministry of Science, Innovation and Universities (MCIU), the State Research Agency (AEI) and the European Regional Development Fund (FEDER), RTI2018-094393-B-C21-MCIU/AEI/FEDER, UE, and the Seneca Foundation CARM, 20786/PI/18. Rosa A. Sola-Martínez is a recipient of an FPU-PhD fellowship from the Ministry of Science, Innovation and Universities (FPU18/00545), and Gema Lozano-Terol is a recipient of a PhD fellowship from Seneca Foundation (20715/FPI/18). Eva Morales was funded by Miguel Servet Fellowships (MS14/00046 and CPII19/00019) awarded by the Instituto de Salud Carlos III (ISCIII), Spanish Ministry of Science, Innovation and Universities, and Fondos FEDER.

The members of the NELA study group are as follows:

ME Candel-Torralba[2], L Garcia-Marcos (PI)[2,4,5,6], MJ Gimenez-Banon[2], A Martinez-Torres[2,4,6], E Morales (PI)[2,3], V Perez-Fernandez[2,5,6], M Sanchez-Solis[2,4,5,6], A Nieto[2,9], MT Prieto-Sanchez[2,9], M Sanchez-Ferrer[2,9], L Fernandez-Palacios[2,10], VP Gomez-Gomez[2,10], C Martinez-Gracia[2,10], P Peso-Echarri[2,10], G Ros-Berruezo[2,10], M Santaella-Pascual[2,10], A Gazquez[2,11], E Larque[2,11], MT Pastor-Fajardo[2,11], M Sanchez-Campillo,[2,11], A Serrano-Munuera[2,11], M Zornoza-Moreno[2,11], P Jimenez-Guerrero[7], E Adomnei[2,3], JJ Arense-Gonzalo[2,3], J Mendiola[2,3], F Navarro-Lafuente[2,3], AM Torres-Cantero[2,3], C Salvador-Garcia[12], M Segovia-Hernández[2,13], G Yagüe-Guirao[2,13], PL Valero-Guillén[2,14], FV Aviles-Plaza[2,8], J Cabezas-Herrera[2,8], A Martinez-Lopez[2,8], M Martinez-Villanueva[2,8], JA Noguera-Velasco[1,2,8], E Cantero-Cano[2], A Franco-Garcia[1,2], AM Garcia-Serna,[1,2], T Hernandez-Caselles[1,2,6], E Martin-Orozco[1,2,6], M Norte-Muñoz[1,2], M Cánovas Díaz[1,2], T de Diego Puente[1,2], JM Pastor[1,2], RA Sola-Martínez[1,2], A Esteban-Gil[2,17], JT Fernández-Breis[2,15], MV Alcántara[16], S Hernández[16], C López-Soler[16]. *[1]Department of Biochemistry and Molecular Biology B and Immunology, University of Murcia, Spain; [2]Biomedical*

Research Institute of Murcia, IMIB-Arrixaca, Murcia, Spain; [3]Department of Public Health Sciences, University of Murcia, Spain; [4]Respiratory and Allergy Units, Arrixaca Children's University Hospital, University of Murcia, Murcia, Spain; [5]Department of Paediatrics, University of Murcia, Spain; [6]Network of Asthma and Adverse and Allergic Reactions (ARADyAL), Health Institute Carlos III, Madrid, Spain; [7]Regional Atmospheric Modelling Group, Department of Physics, University of Murcia, Spain; [8]Molecular Therapy and Biomarkers Research Group, Clinical Analysis Service, University Clinical Hospital "Virgen de la Arrixaca", University of Murcia, Spain; [9]Obstetrics & Gynaecology Service, "Virgen de la Arrixaca" University Clinical Hospital, University of Murcia, Spain; [10]Food Science and Technology Department, Veterinary Faculty of Veterinary, University of Murcia, Spain; [11]Department of Physiology, Faculty of Biology, Campus Mare Nostrum. University of Murcia, Spain; [12]Microbiology Service, General University Hospital Consortium, University of Valencia, Spain; [13]Microbiology Service, University Clinical Hospital "Virgen de la Arrixaca", University of Murcia, Spain; [14]Microbiology and Genetics Department, University of Murcia, Spain; [15]Department of Informatics and Systems, University of Murcia, Spain; [16]Paediatric and Adolescent Clinical Psychology University Research Group (GUIIA-PC), University of Murcia, Spain; [17]Foundation for Healthcare Training & Research of the Region of Murcia (FFIS).

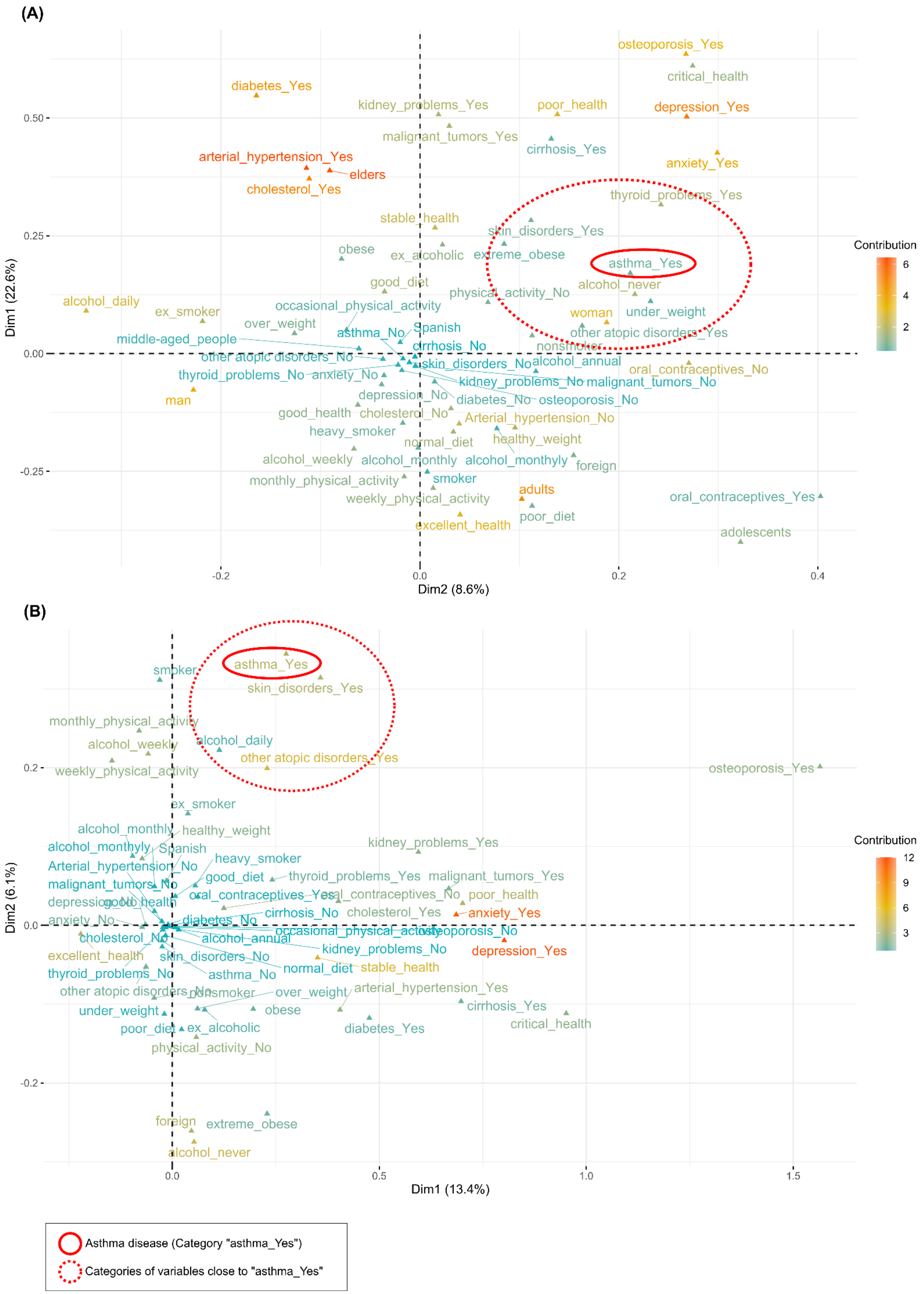

Supplemental Figure S1. Representation of MCA (multiple correspondence analysis) results performed on data collection from the Spanish National Health Survey of 2017 (ENSE-2017). A) All data collected from the ENSE-2017. B) Open cohort of 18- to 45-year-old women constructed using the ENSE-2017

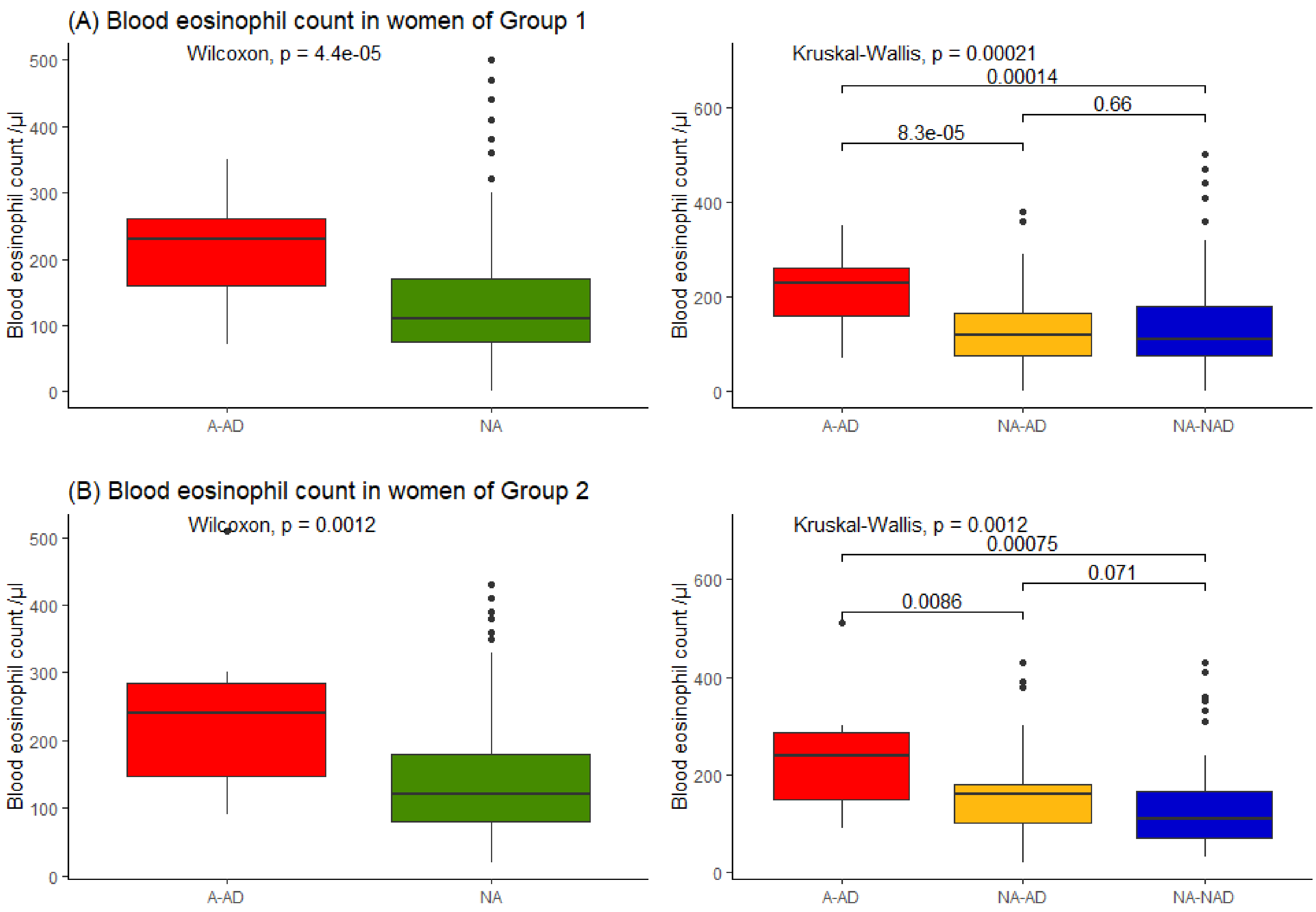

Supplementary Figure S2. Blood eosinophil count in women of childbearing age. A) Blood eosinophil count in women of Group 1. B) Blood eosinophil count in women of Group 2. A-AD: asthmatics with other coexisting atopic diseases; NA: non-asthmatics; NA-NAD: non-asthmatics without atopic diseases; NA-AD: Non-asthmatics with other atopic diseases.

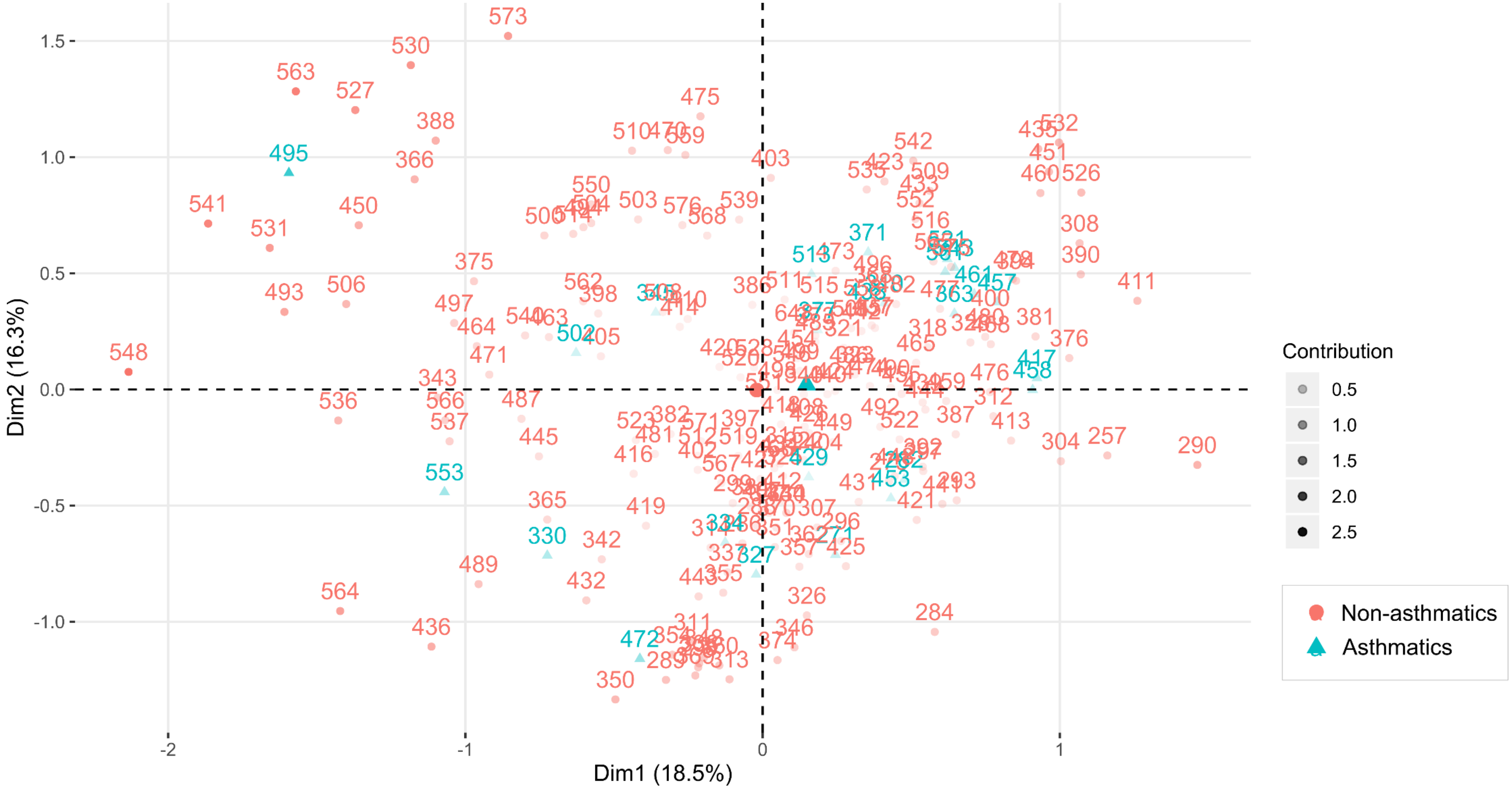

Supplementary Figure S3. Exploratory analysis of asthma disease influence in exhaled breath of women. Representation PCA (principal component analysis) results conducted on filtered features from exhaled breath of women of Group 1 after removing pollutant features (hyper-filtered features).

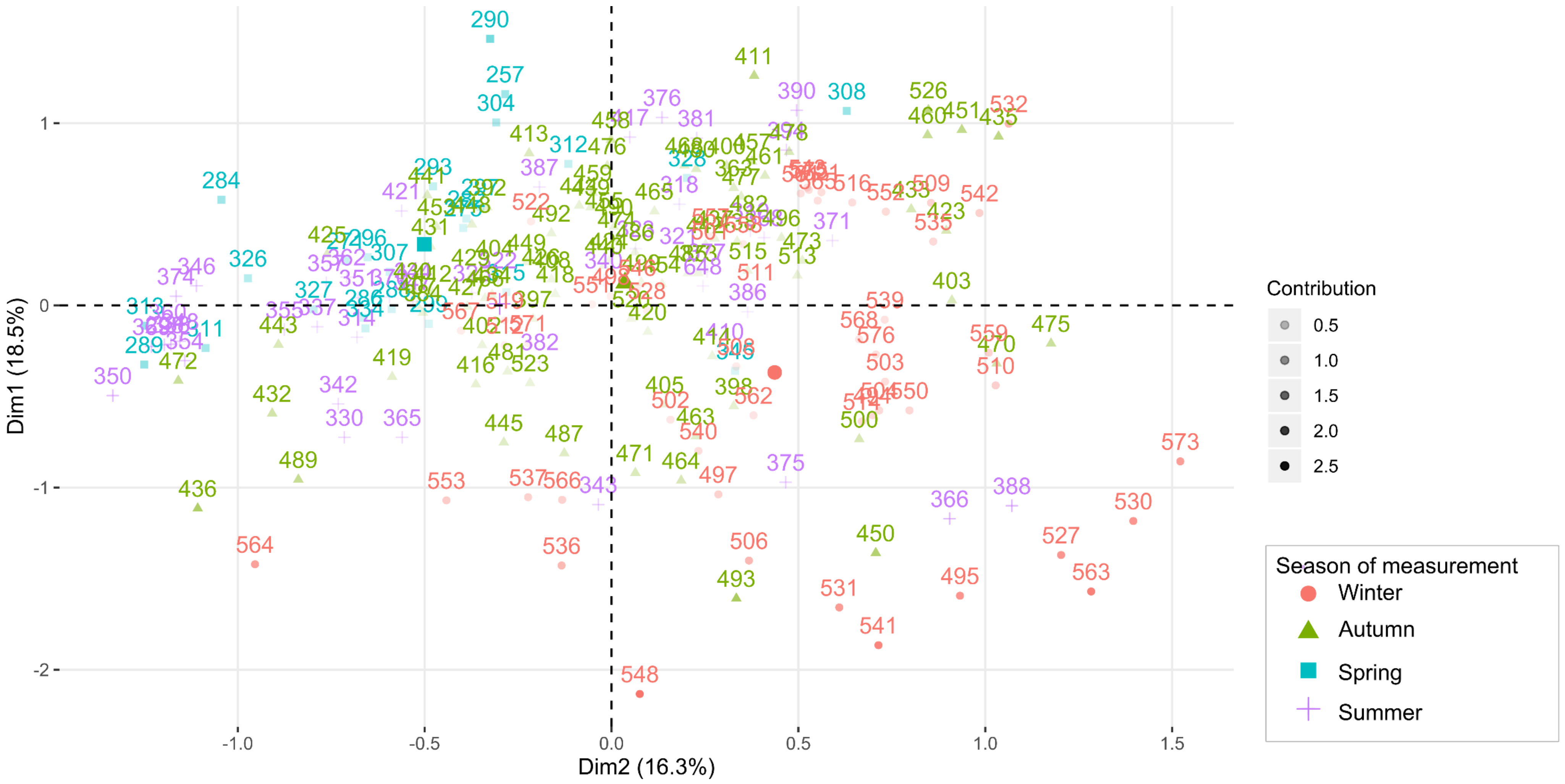

Supplementary Figure S4. Exploratory analysis of seasonal variation in sampling of exhaled breath of women. Representation PCA (principal component analysis) results conducted on filtered features from exhaled breath of women of Group 1 after removing pollutant features (hyper-filtered features).

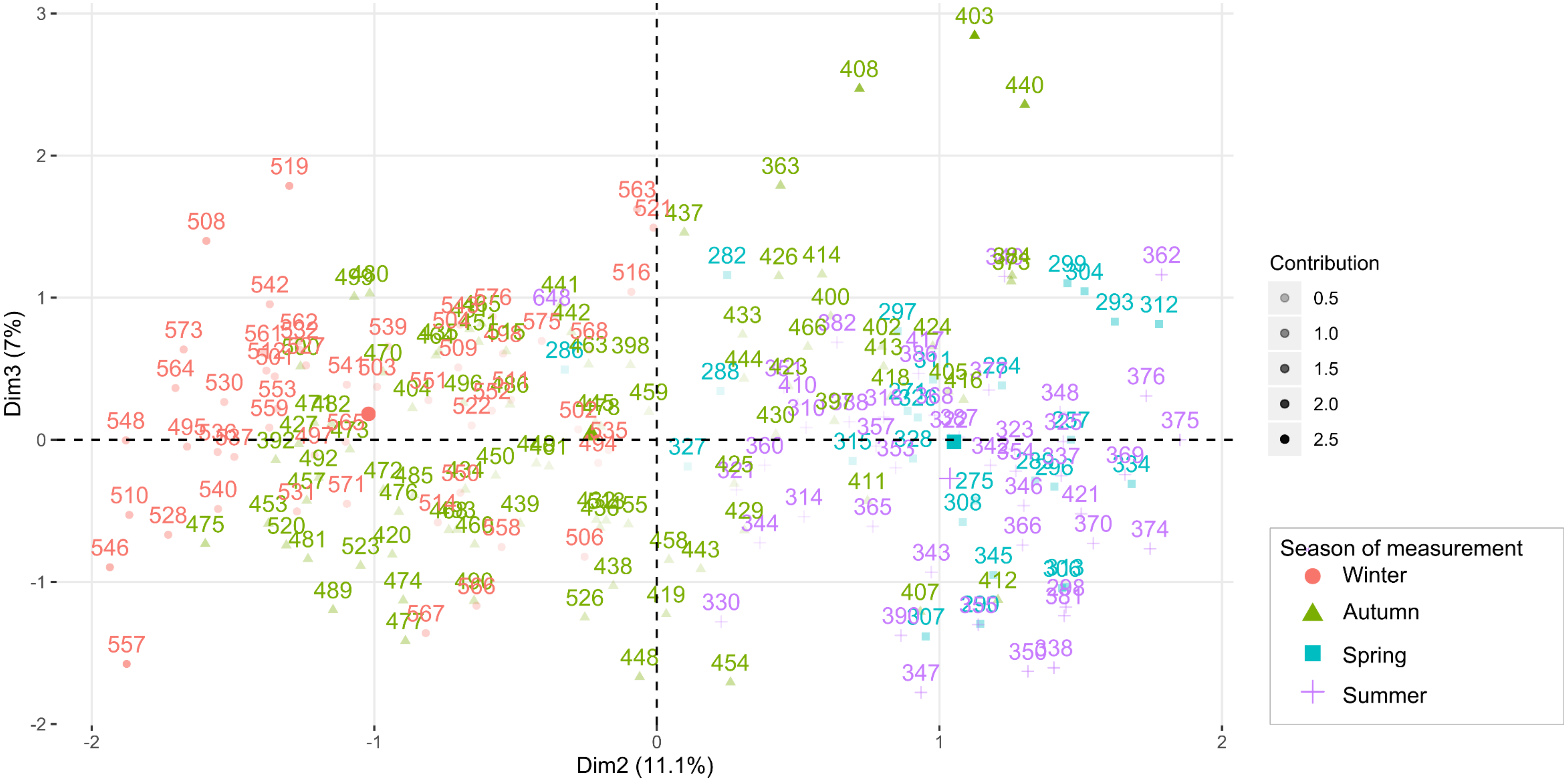

Supplementary Figure S5. Exploratory analysis of seasonal variation in sampling of ambient air samples. Representation PCA (principal component analysis) results conducted on filtered features from room air content samples of Group 1.

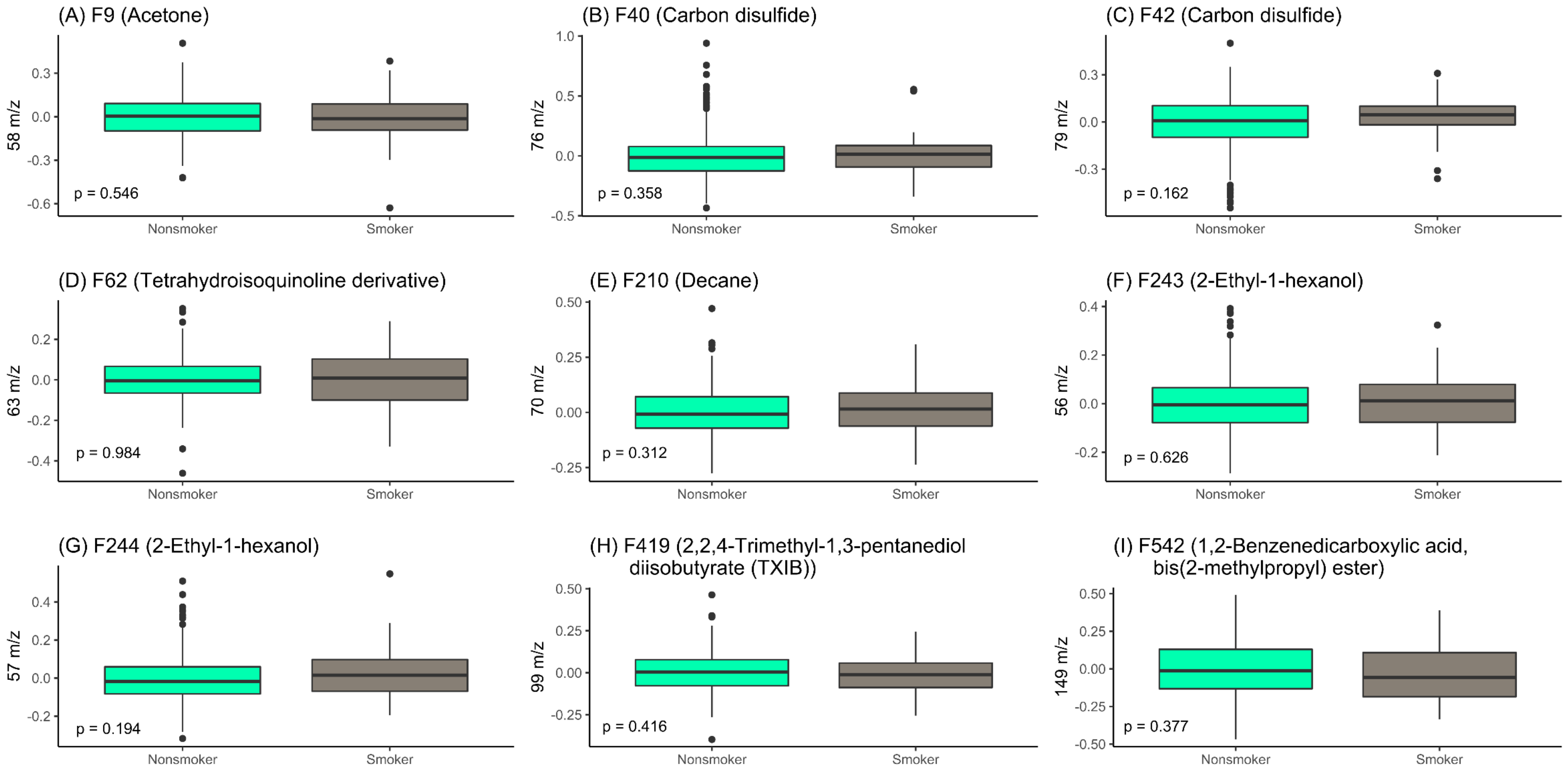

Supplementary Figure S6. Assessment of smoking habits influence on the levels in exhaled breath of the features selected as discriminants in the significant models (model I.A, model I.B, model I.C, model I.D, model I.E, model I.F, model I.G, model II.A, model II.B, model II.C, model II.D, model III.A, model III.B, model III.C and model III.D). A) Impact of smoking habits on levels of acetone (F9) in exhaled breath. B) Impact of smoking habits on levels of carbon disulfide (F40) in exhaled breath. C) Impact of smoking habits on levels of carbon disulfide (F42) in exhaled breath. D) Impact of smoking habits on levels of tetrahydroisoquinoline derivative (F62) in exhaled breath. E) Impact of smoking habits on levels of decane (F210) in exhaled breath. F) Impact of smoking habits on levels of 2-ethyl-1-hexanol (F243) in exhaled breath. G) Impact of smoking habits on levels of 2-ethyl-1-hexanol (F244) in exhaled breath. H) 2,2,4-Trimethyl-1,3-pentanediol diisobutyrate (TXIB) (F419). I) 1,2-Benzenedicarboxylic acid, bis(2-methylpropyl) ester (F542).

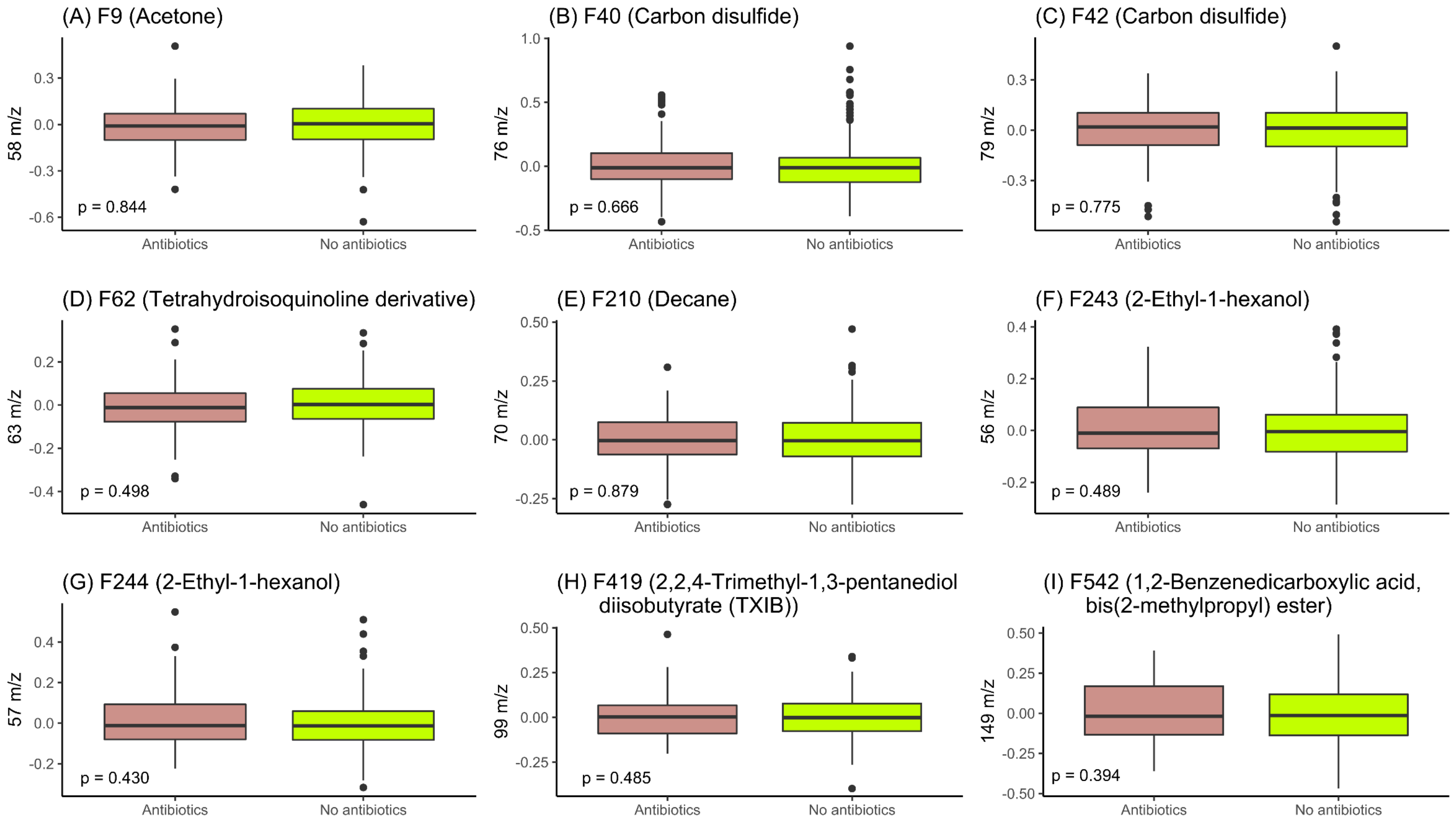

Supplementary Figure S7. Assessment of antibiotics consumption influence on the levels in exhaled breath of the features selected as discriminants in the significant models (model I.A, model I.B, model I.C, model I.D, model I.E, model I.F, model I.G, model II.A, model II.B, model II.C, model II.D, model III.A, model III.B, model III.C and model III.D). A) Impact of antibiotics consumption on levels of acetone (F9) in exhaled breath. B) Impact of antibiotics consumption on levels of carbon disulfide (F40) in exhaled breath. C) Impact of antibiotics consumption on levels of carbon disulfide (F42) in exhaled breath. D) Impact of antibiotics consumption on levels of tetrahydroisoquinoline derivative (F62) in exhaled breath. E) Impact of antibiotics consumption on levels of decane (F210) in exhaled breath. F) Impact of antibiotics consumption on levels of 2-ethyl-1-hexanol (F243) in exhaled breath. G) Impact of antibiotics consumption on levels of 2-ethyl-1-hexanol (F244) in exhaled breath. H) 2,2,4-Trimethyl-1,3-pentanediol diisobutyrate (TXIB) (F419). I) 1,2-Benzenedicarboxylic acid, bis(2-methylpropyl) ester (F542).

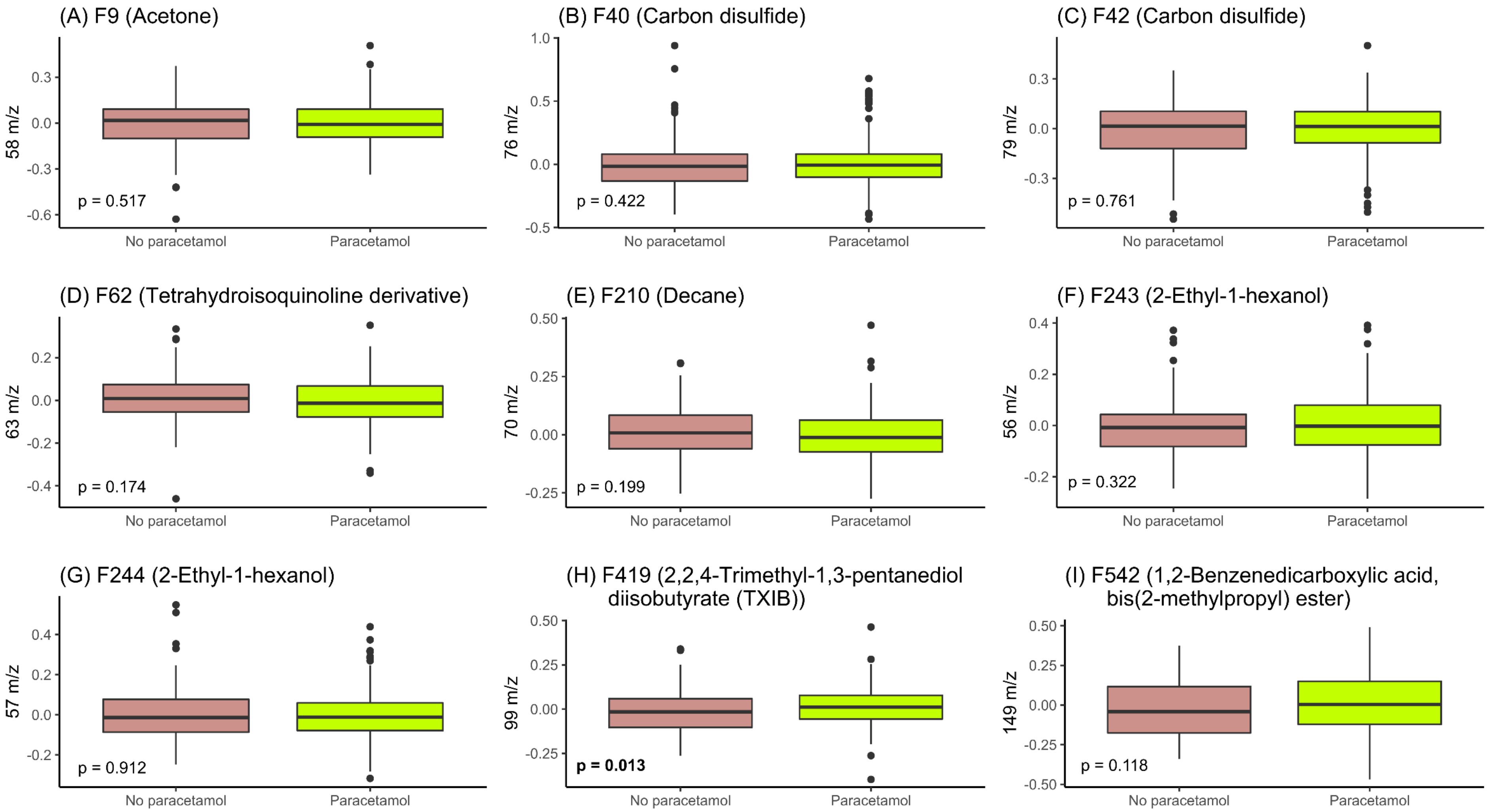

Supplementary Figure S8. Assessment of paracetamol consumption influence on the levels in exhaled breath of the features selected as discriminants in the significant models (model I.A, model I.B, model I.C, model I.D, model I.E, model I.F, model I.G, model II.A, model II.B, model II.C, model II.D, model III.A, model III.B, model III.C and model III.D). A) Impact of paracetamol consumption on levels of acetone (F9) in exhaled breath. B) Impact of paracetamol consumption on levels of carbon disulfide (F40) in exhaled breath. C) Impact of paracetamol consumption on levels of carbon disulfide (F42) in exhaled breath. D) Impact of paracetamol consumption on levels of tetrahydroisoquinoline derivative (F62) in exhaled breath. E) Impact of paracetamol consumption on levels of decane (F210) in exhaled breath. F) Impact of paracetamol consumption on levels of 2-ethyl-1-hexanol (F243) in exhaled breath. G) Impact of paracetamol consumption on levels of 2-ethyl-1-hexanol (F244) in exhaled breath. H) 2,2,4-Trimethyl-1,3-pentanediol diisobutyrate (TXIB) (F419). I) 1,2-Benzenedicarboxylic acid, bis(2-methylpropyl) ester (F542).

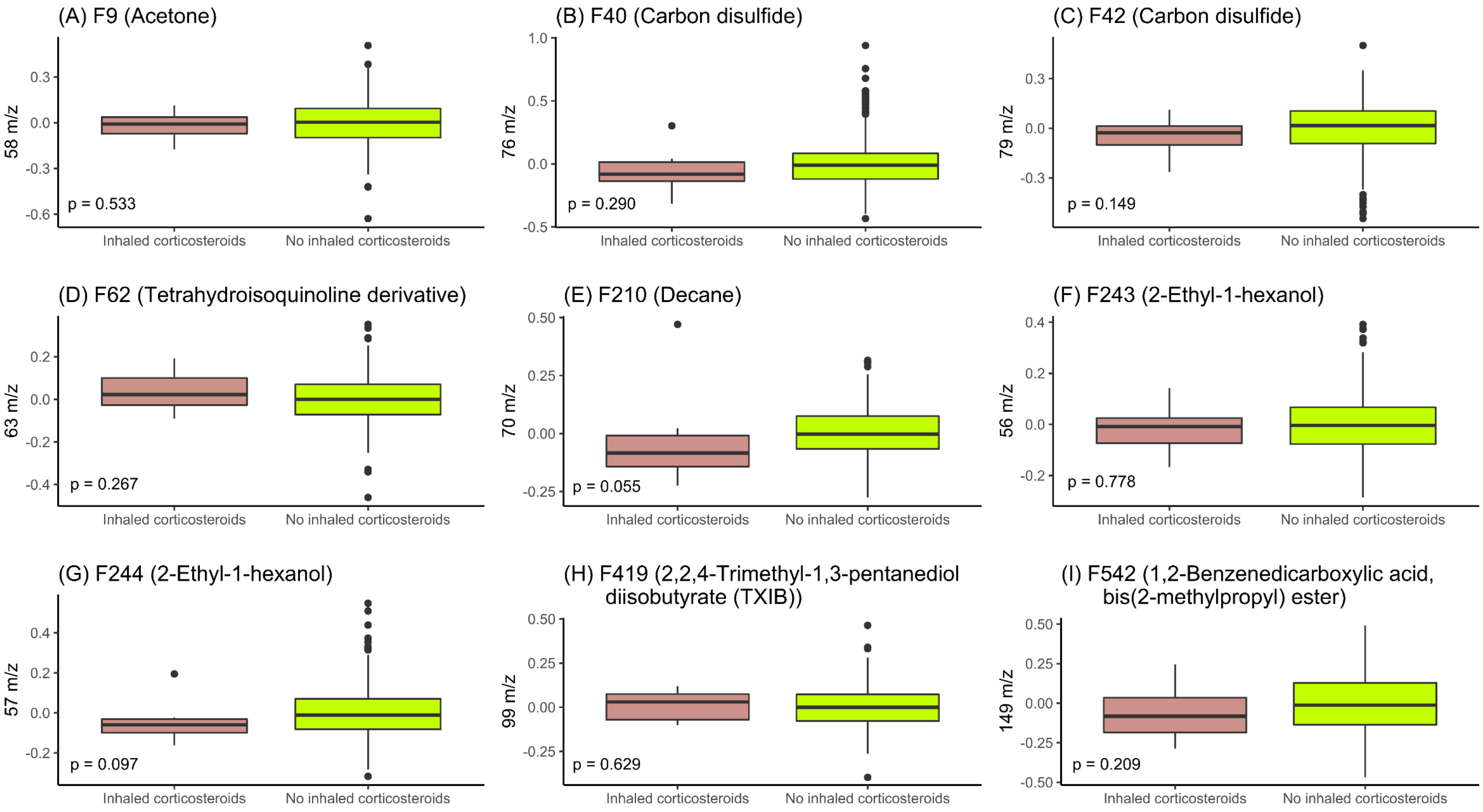

Supplementary Figure S9. Assessment of inhaled corticosteroids consumption influence on the levels in exhaled breath of the features selected as discriminants in the significant models (model I.A, model I.B, model I.C, model I.D, model I.E, model I.F, model I.G, model II.A, model II.B, model II.C, model II.D, model III.A, model III.B, model III.C and model III.D). A) Impact of inhaled corticosteroids consumption on levels of acetone (F9) in exhaled breath. B) Impact of inhaled corticosteroids consumption on levels of carbon disulfide (F40) in exhaled breath. C) Impact of inhaled corticosteroids consumption on levels of carbon disulfide (F42) in exhaled breath. D) Impact of inhaled corticosteroids consumption on levels of tetrahydroisoquinoline derivative (F62) in exhaled breath. E) Impact of inhaled corticosteroids consumption on levels of decane (F210) in exhaled breath. F) Impact of inhaled corticosteroids consumption on levels of 2-ethyl-1-hexanol (F243) in exhaled breath. G) Impact of inhaled corticosteroids consumption on levels of 2-ethyl-1-hexanol (F244) in exhaled breath. H) 2,2,4-Trimethyl-1,3-pentanediol diisobutyrate (TXIB) (F419). I) 1,2-Benzenedicarboxylic acid, bis(2-methylpropyl) ester (F542).

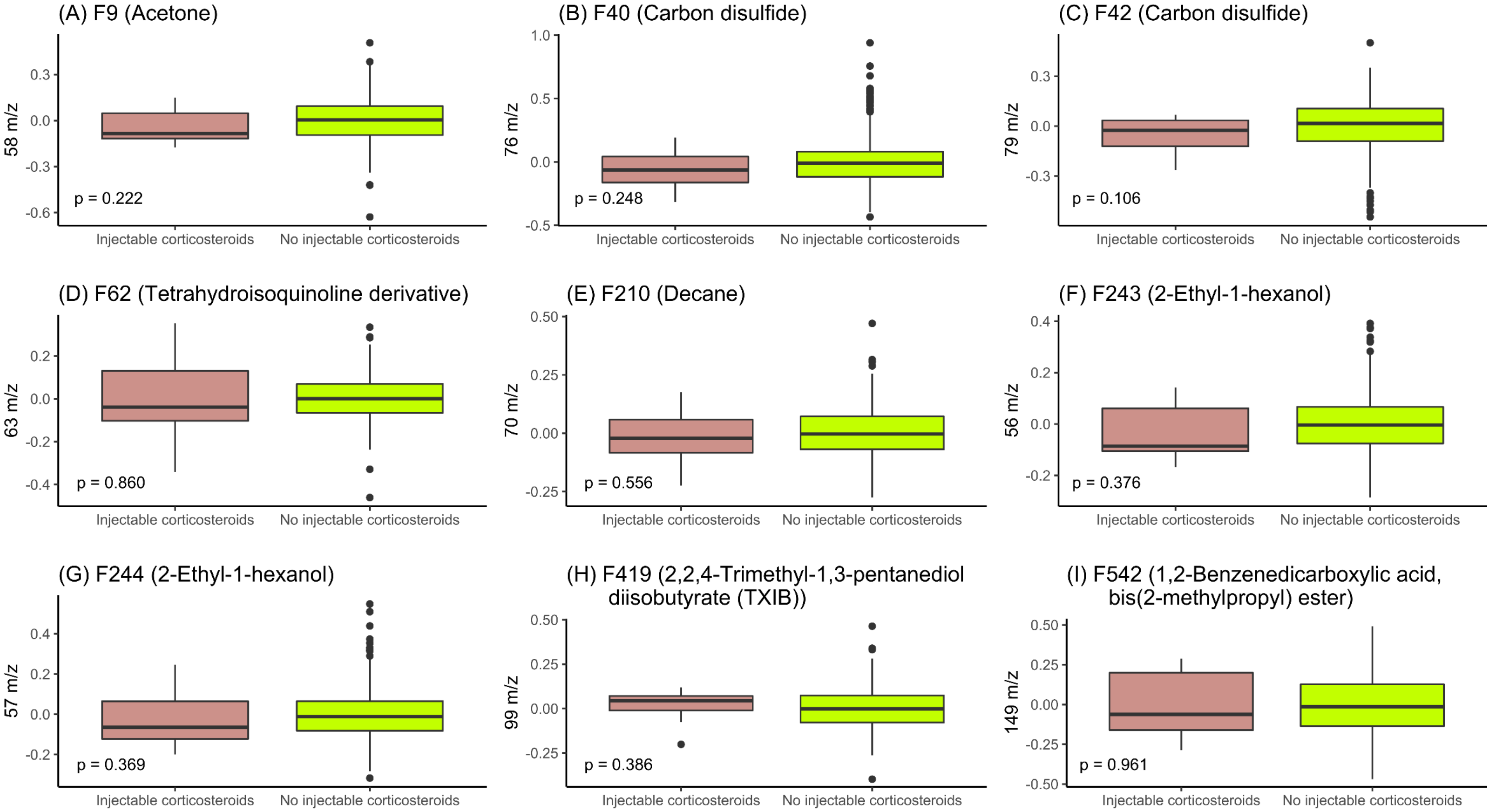

Supplementary Figure S10. Assessment of injectable corticosteroids consumption influence on the levels in exhaled breath of the features selected as discriminants in the significant models (model I.A, model I.B, model I.C, model I.D, model I.E, model I.F, model I.G, model II.A, model II.B, model II.C, model II.D, model III.A, model III.B, model III.C and model III.D). A) Impact of injectable corticosteroids consumption on levels of acetone (F9) in exhaled breath. B) Impact of injectable corticosteroids consumption on levels of carbon disulfide (F40) in exhaled breath. C) Impact of injectable corticosteroids consumption on levels of carbon disulfide (F42) in exhaled breath. D) Impact of injectable corticosteroids consumption on levels of tetrahydroisoquinoline derivative (F62) in exhaled breath. E) Impact of injectable corticosteroids consumption on levels of decane (F210) in exhaled breath. F) Impact of injectable corticosteroids consumption on levels of 2-ethyl-1-hexanol (F243) in exhaled breath. G) Impact of injectable corticosteroids consumption on levels of 2-ethyl-1-hexanol (F244) in exhaled breath. H) 2,2,4-Trimethyl-1,3-pentanediol diisobutyrate (TXIB) (F419). I) 1,2-Benzenedicarboxylic acid, bis(2-methylpropyl) ester (F542).

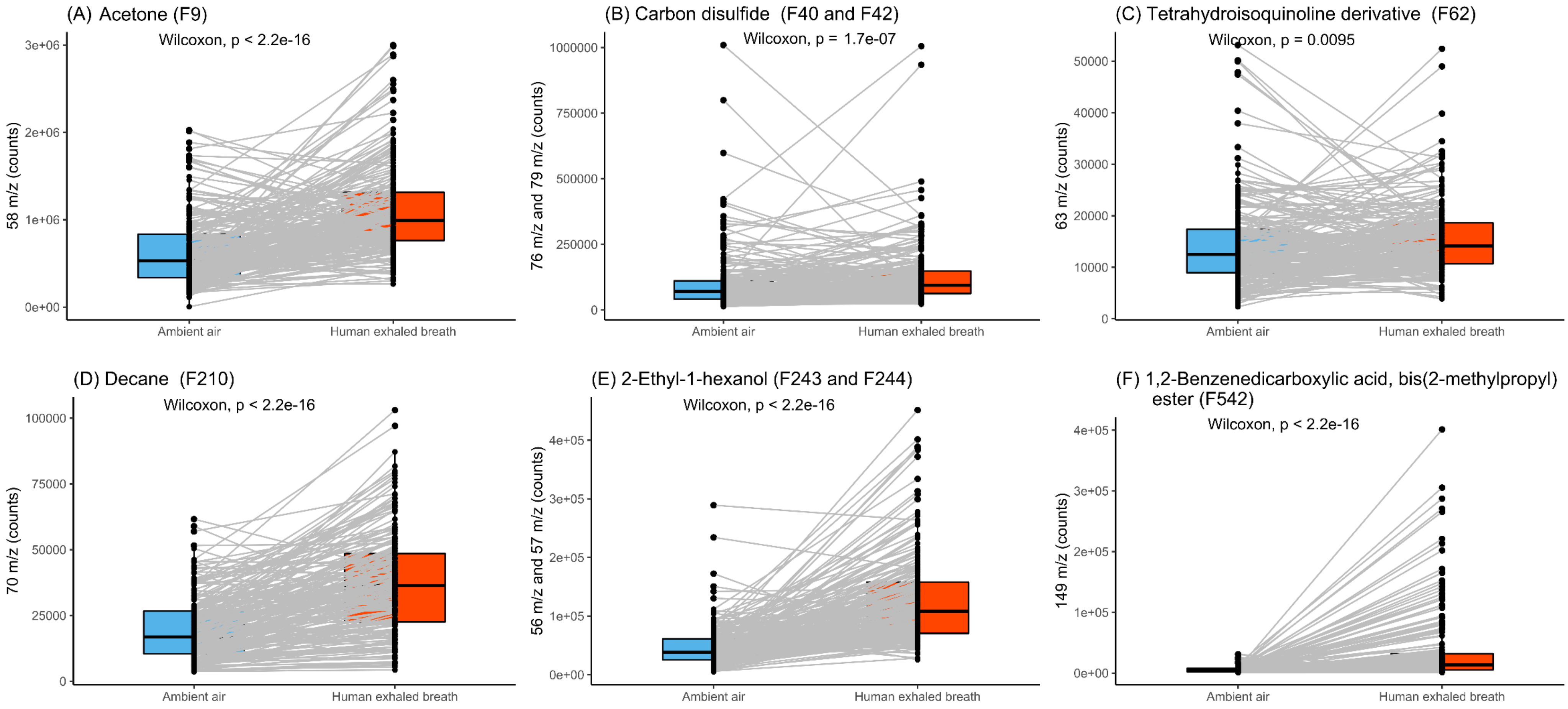

Supplementary Figure S11. Comparison of the intensities of features from discriminant VOCs in environmental samples and human exhaled breath samples from the NELA cohort. 2,2,4-Trimethyl-1,3-pentanediol diisobutyrate (F419) was not detected in room air content samples. A) Intensity of acetone (F9) in ambient air and human exhaled breath. B) Intensity of carbon disulfide (F40-F42) in ambient air and human exhaled breath. C) Intensity of tetrahydroisoquinoline derivative (F62) in ambient air and human exhaled breath. D) Intensity of decane (F210) in ambient air and human exhaled breath. E) Intensity of 2-ethyl-1-hexanol (F243) in ambient air and human exhaled breath. F) Intensity of 1,2-Benzenedicarboxylic acid, bis(2-methylpropyl) ester (F542) in ambient air and human exhaled breath.

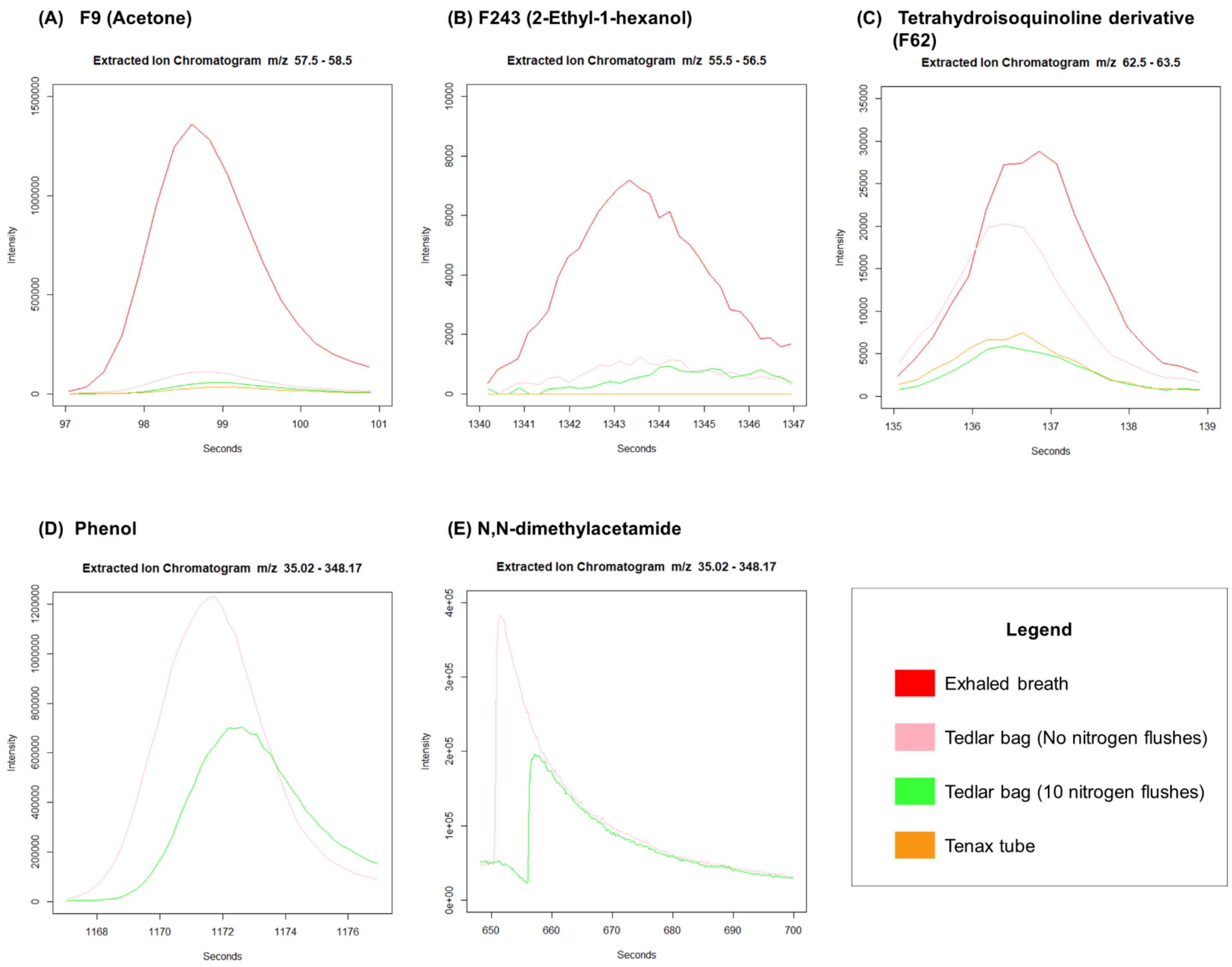

Supplementary Figure S12. Tedlar bags background artifacts and Tenax degradation products influence in intensities of features of VOCs selected as discriminants of asthma with other coexisting atopic diseases (acetone, 2-ethyl-1-hexanol and a tetrahydroisoquinoline derivative). A) Intensity of feature F9 (acetone) in exhaled breath, in Tedlar bag and in a reconditioned Tenax tube. B) Intensity of feature F243 (2-ethyl-1-hexanol) in exhaled breath, in Tedlar bag and in a reconditioned Tenax tube. C) Intensity of feature F62 (tetrahydroisoquinoline) in exhaled breath, in Tedlar bag and in a reconditioned Tenax tube. D) Phenol intensity in the Tedlar bag before and after nitrogen flushes. E) N,N-dimethylacetamide intensity in the Tedlar bag before and after nitrogen flushes.

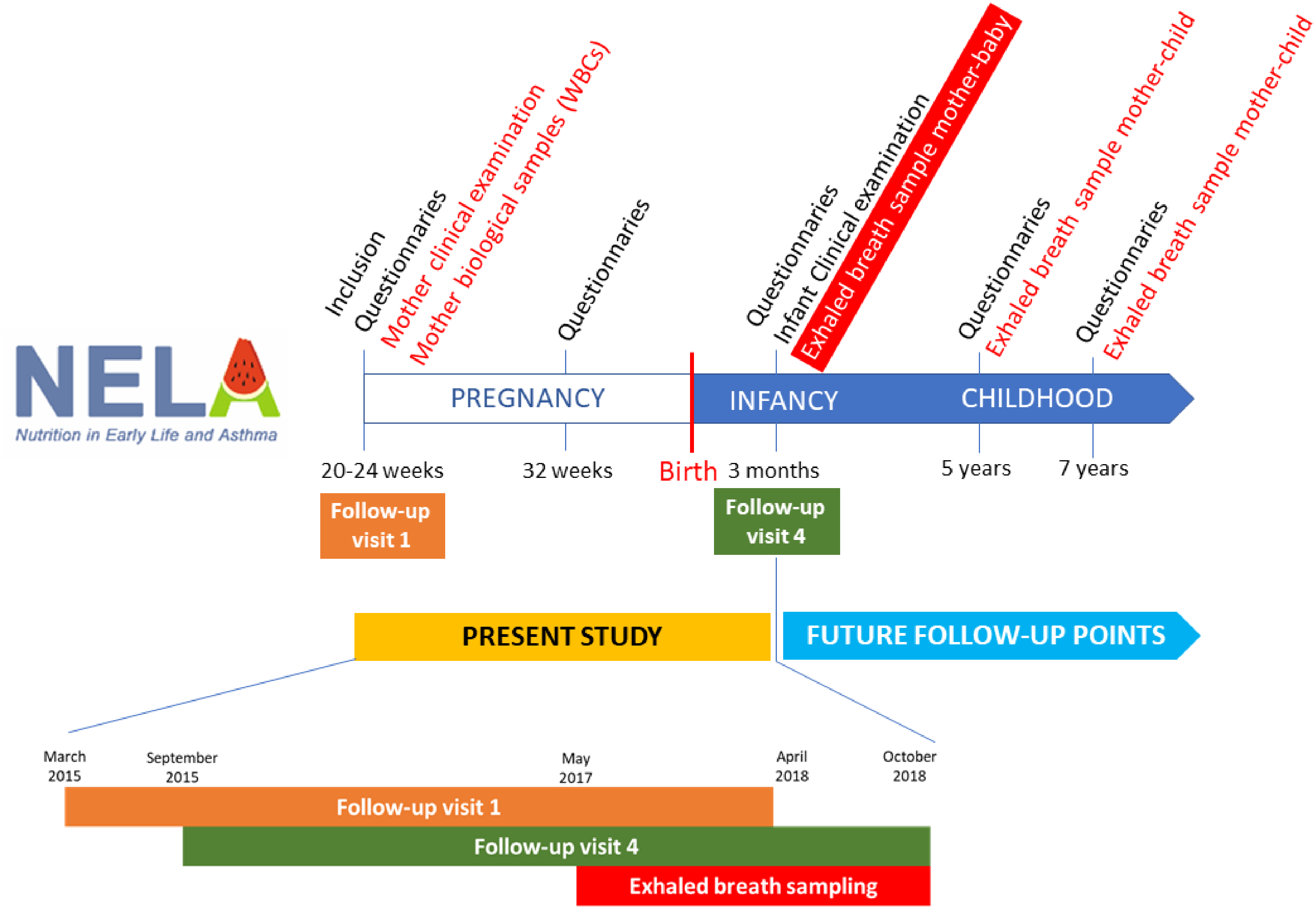

Supplementary Figure S13. Diagram of the follow-up points of the subjects enrolled in the NELA (Nutrition in Early Life and Asthma) cohort. The present study is embedded in the NELA study.

Supplemental Table S1. Analysis of associations between asthma and variables listed in EHSS-2014 (European Health Survey in Spain 2014) and ENSE-2017 (Spanish National Health Survey of 2017).

		European Health Survey for Spain 2014		Spanish National Health Survey of 2017	
		All subjects	Only women of childbearing age	All subjects	Only women of childbearing age
**Variables**	**Variable categories**	**p-value**	**p-value**	**p-value**	**p-value**
**Age**	-Children (15-18 years)<br>-Adults (18-45 years)<br>-Middle-aged people (45-65 years)<br>-Elders (>65 years)	**8.827e-06**	-	**1.689e-06**	-
**Gender**	-Man<br>-Woman	**1.291e-05**	-	**3.041e-07**	-
**Nationality**	-Spanish<br>-Foreign	0.072	**0.037**	**0.026**	0.078
**Health status**	-Excellent health<br>-Good health<br>-Stable health<br>-Poor health<br>-Critical health	**< 2.2e-16**	**6.344e-12**	**< 2.2e-16**	**1.587e-09**
**Arterial hypertension**	-Arterial hypertension_Yes<br>-Arterial hypertension_No	**1.472e-05**	**0.024**	**0.002**	**6.081e-04**
**Other atopic disorders (any atopic disease excluding asthma)**	-Other atopic disorders_Yes<br>-Other atopic disorders_No	**< 2.2e-16**	**< 2.2e-16**	**< 2.2e-16**	**< 2.2e-16**
**Diabetes**	-Diabetes_Yes<br>-Diabetes_No	**8.113e-05**	0.792	**5.024e-07**	0.301
**Cholesterol**	-Cholesterol_Yes<br>-Cholesterol_No	0.126	**0.010**	**0.002**	0.375
**Skin disorders**	-Skin disorders_Yes<br>-Skin disorders_No	**1.056e-14**	**5.586e-06**	**< 2.2e-16**	**5.299e-06**
**Cirrhosis**	-Cirrhosis_Yes<br>-Cirrhosis_No	**0.008**	0.122	**0.041**	0.321
**Depression**	-Depression_Yes<br>-Depression_No	**< 2.2e-16**	0.075	**< 2.2e-16**	**0.041**
**Anxiety disorders**	-Anxiety_Yes<br>-Anxiety_No	**< 2.2e-16**	**6.547e-05**	**1.961e-14**	**0.024**
**Kidney problems**	-Kidney problems_Yes<br>-Kidney problems_No	**6.601e-06**	0.135	**7.152e-08**	0.387
**Malignant tumors**	-Malignant tumors_Yes<br>-Malignant tumors_No	0.122	0.104	**1.118e-04**	0.424
**Thyroid problems**	-Thyroid problems_Yes<br>-Thyroid problems_No	**5.288e-07**	0.146	**0.001**	0.812
**Osteoporosis**	-Osteoporosis_Yes<br>-Osteoporosis_No	**5.217e-08**	1	**2.216e-12**	0.262
**Oral contraceptives**	-Oral_contraceptives_Yes<br>-Oral_contraceptives_No	0.205	0.476	0.841	0.917
**BMI**	-Under weight<br>-Healthy weight<br>-Over weight<br>-Obese<br>-Extreme obese	**5.963e-08**	**3.231e-04**	**2.197e-11**	0.506
**Physical activity**	-Physical activity_No<br>-Occasional physical activity<br>-Monthly physical activity<br>-Weekly physical activity	**6.965e-05**	0.942	0.095	0.093
**Diet**	-Poor diet<br>-Normal diet<br>-Good diet	0.281	**0.037**	0.240	0.693
**Tobacco**	-Heavy smoker<br>-Smoker<br>-Ex-smoker<br>-Non-smoker	**0.006**	0.630	**8.206e-05**	0.342
**Alcohol**	-Alcohol daily<br>-Alcohol weekly<br>-Alcohol monthly<br>-Alcohol annual<br>-Ex-alcoholic<br>-Alcohol never	0.092	0.122	**0.003**	**0.004**

Supplementary Table S2. Formulas of the constructed models.

Models	Predicted probability (y)
**I) A-AD vs NA**	
**I.A**	y= 1/ (1+exp(−(0.014∗F9+1.232∗F62+1.894∗F243−2.042)))
**I.B**	y= 1/ (1+exp(-(0.388∗F9+1.526∗F62+1.861∗F243+0.840∗ Parental asthma +0.261∗ Parental rhinitis +0.164∗ Parental dermatitis−2.217)))
**I.C**	y= 1/ (1+exp(-(0.138∗F62+0.845∗F243+3.171∗ Blood eosinophil count−2.498)))
**I.D**	y= 1/ (1+exp(-(0.687∗F62+1.200∗F243+3.478∗ Blood eosinophil count +0.552∗ Parental asthma +0.160∗ Parental rhinitis−2.660)))
**I.E**	y= 1/ (1+exp(-(0.038∗F42+0.724∗F62 +1.060∗F243+1.777*Allergic rhinitis+0.653*Atopic dermatitis+0.646*Allergic conjuntivitis+0.337*Food allergy+0.348*Drug allergy−3.362)))
**I.F**	y= 1/ (1+exp(-(0.216*F9−0.296∗F40+0.231*F42+1.134∗F62 +1.388∗F243+0.579∗ Parental asthma +0.272∗Parental dermatitis+ 1.872*Allergic rhinitis+0.675*Atopic dermatitis+0.652*Allergic conjuntivitis+0.473*Food allergy+0.530*Drug allergy−3.582)))
**I.G**	y= 1/ (1+exp(-(−0.356∗F40+0.981∗F62 +0.881∗F243 +0.421∗Parental asthma + 0.126∗ Parental dermatitis+4.287∗ Blood eosinophil count +1.892*Allergic rhinitis+0.609*Atopic dermatitis+0.599*Allergic conjuntivitis+0.703*Food allergy+0.541*Drug allergy −4.232)))
**II) A-AD vs NA-NAD**	
**II.A**	y= 1/ (1+exp(-(0.282∗F9+0.789∗F62+1.292∗F243−1.544)))
**II.B**	y= 1/ (1+exp(-(0.648∗F9+1.292∗F62+1.754∗F243+0.809∗ Parental asthma +0.694∗Parental rhinitis −1.799)))
**II.C**	y= 1/ (1+exp(-(0.036*F9+0.503∗F62+1.157∗F243+2.660∗ Blood eosinophil count−1.955)))
**II.D**	y= 1/ (1+exp(-(0.0004*F9+0.582∗F62+1.233∗F243+2.431∗ Blood eosinophil count +0.562∗ Parental asthma +0.605∗ Parental rhinitis−2.101)))
**III) A-AD vs NA-AD**	
**III.A**	y= 1/ (1+exp(-(−0.301∗F40+0.267∗F42+0.683∗F62+0.287∗F210+1.232∗F243+0.144∗F419 −1.077)))
**III.B**	y= 1/ (1+exp(-(−0.829∗F40+0.472*F42+1.245∗F62+0.589∗F210+1.760∗F243+0.100*F419+0.104∗ Parental asthma +0.146∗ Parental dermatitis−1.099)))
**III.C**	y= 1/ (1+exp(-(−0.827∗F40+1.148∗F62+0.420∗F210+0.925∗F243+0.124∗F542+6.886∗ Blood eosinophil count −0.155∗ Blood lymphocyte count −1.885)))
**III.D**	y= 1/ (1+exp(-(−1.379∗F40+1.613∗F62+0.700∗F210+1.439∗F243+0.008∗F244+0.433∗F542+7.614∗ Blood eosinophil count −0.264∗ Blood lymphocyte count +0.034∗ Parental asthma +0.398∗ Parental dermatitis−1.846)))
**IV) NA-AD vs NA-NAD**	
**IV.A**	y= 1/ (1+exp(-(−0.201∗F23+0.461∗F360−0.65∗F421 −0,461)))

A-AD: asthmatics with other coexisting atopic diseases; NA: non-asthmatics; NA-NAD: non-asthmatics without atopic diseases; NA-AD: Non-asthmatics with other atopic diseases.

Supplementary Table S3. Characteristics of features selected by the constructed models.

Features	m/z	Models	VOC	CAS number	Match factor and error RI values	Only Match factor value
**F9**	58	I.A, I.B, I.F, II.A, II.B, II.C, II.D	Acetone	67-64-1	X	
**F23**	50	IV.A	Isoprene	78-79-5		X
**F40**	76	I.F, I.G, III.A, III.B, III.C, III.D	Carbon disulfide	75-15-0	X	
**F42**	79	I.E, III.A, III.B				
**F62**	63	I.A, I.B, I.C, I.D, I.E, I.F, I.G, II.A, II.B, II.C, II.D, III.A, III.B, III.C, III.D	Tetrahydroisoquinoline derivative			X
**F210**	70	III.A, III.B, III.C, III.D	Decane	124-18-5	X	
**F243**	56	I.A, I.B, I.C, I.D, I.E, I.F, I.G, II.A, II.B, II.C, II.D, III.A, III.B, III.C, III.D	2-Ethyl-1-hexanol	104-76-7	X	
**F244**	57	III.D				
**F360**	118	IV.A	2-Propenoic acid, 3-(2-hydroxyphenyl)-, (E)-	614-60-8		X
**F419**	99	III.A, III.B	2,2,4-Trimethyl-1,3-pentanediol diisobutyrate (TXIB)	6846-50-0	X	
**F421**	57	IV.A				
**F542**	149	III.C, III.D	1,2-Benzenedicarboxylic acid, bis(2-methylpropyl) ester	84-69-5	X	

Error RI: retention index error.
